# Engineering human endogenous retrovirus-K for improving immunogenicity and broad cancer targeting

**DOI:** 10.1186/s12967-026-09012-1

**Published:** 2026-09-24

**Authors:** Mikkel Dons Müller, Lasse Neukirch, Emeline Ragonnaud, Amaia Vergara Bermejo, Joana Daradoumis, Victoria Jaded, Emilie Sofie Engdal, Felix Faas, Maria Perez-Penco, Cristina Picon-Jara, Karen Nørgaard Nielsen, Lara Duvnjak, Ditte Rahbaek Boilesen, Sara Kusiński, Isabella Skandorff, Nanna Skeltved, Lene Linh Ngoc Trinh, Louise Turner, Kaare Villum Grunddal, Huda Salahudin Darusman, Silmi Mariya, Silke Schrödel, Kristoffer Staal Rohrberg, Frederik Otzen Bagger, Christian Thirion, Per Thor Straten, Anne-Marie Carola Andersson, Peter Johannes Holst

**Affiliations:** 1 Department of Immunology and Microbiology, University of Copenhagen, Copenhagen, Denmark; 2 HERVolution Therapeutics ApS, Frederiksberg, Denmark; 3 Department of Biomedical Sciences, University of Copenhagen, Copenhagen, Denmark; 4 Department of Veterinary Biomedical Sciences, School of Veterinary Medicines and Biomedical Sciences, IPB University, Bogor, Indonesia; 5 Primate Research Center, IPB University, Bogor, Indonesia; 6 Revvity Gene Delivery GmbH, Gräfelfing, Germany; 7 Department of Clinical Medicine, University of Copenhagen, Copenhagen, Denmark; 8 Department of Oncology, Copenhagen University Hospital, Rigshospitalet, Copenhagen, Denmark; 9 Department of Genomic Medicine, Copenhagen University Hospital, Rigshospitalet, Copenhagen, Denmark; 10 National Center for Cancer Immune Therapy, Herlev Hospital, Herlev, Denmark

**Keywords:** Immunotherapy, Cancer, Endogenous retrovirus, Adenoviral vectors, HERV-K

## Abstract

**Background:**

HERV-K (HML-2) is the most studied transcriptionally active endogenous retrovirus in human cancers, yet immune tolerance and incomplete mechanistic understanding have impeded its clinical use as an immunotherapy target. We hypothesized that rational engineering of the HERV-K envelope putative immunosuppressive domain (ISD) could improve immunogenicity.

**Methods:**

We performed a mutational screen across the ISD region, evaluated NF-κB activation and then analyzed antigen expression in human monocyte-derived dendritic cells (moDCs) using flow cytometry, RNA-seq, and TEM. Human PBMCs were expanded with HERV-K–transduced moDCs to assess T cell priming and expansion ex vivo. Immunogenicity was tested in mice, HERV-K transgenic mice, and cynomolgus macaques using adenoviral and LNP-mRNA vectors. Antitumor efficacy was evaluated in a murine metastatic renal carcinoma HERV-K expressing tumor model.

**Results:**

Engineering the HERV-K envelope ISD to Q525A (ISDmut) enhanced NF-κB activation, antigen expression, and antigen presentation and cell death associated gene expression pathways in human dendritic cells while preserving virus-like particle assembly. HERV-K-ISDmut-transduced human dendritic cells expanded CD4^+^ and CD8^+^ T cells capable of recognizing HLA-matched, HERV-K expressing tumors, demonstrating that T cells from the human repertoire can be expanded ex vivo. In vivo, mRNA and adenoviral vectors encoding HERV-K-ISDmut elicited strong immune responses in mice, broke tolerance in HERV-K transgenic mice, and induced humoral responses in cynomolgus macaques. In mice, an adenovirus vectored HERV-K-ISDmut immunization further conferred increased survival in a murine renal carcinoma HERV-K expressing tumor model and benefitted from anti-PD-1 therapy.

**Conclusions:**

These results show that the rational redesign of the HERV-K ISD generates a potent immunogen capable of expanding human T cells and mediating mechanistically relevant anti-tumor immunity. We propose that HERV-K-ISDmut antigens represent a promising platform for cancer immunotherapy which should be explored in future clinical trials.

**Supplementary Information:**

The online version contains supplementary material available at https://doi.org/10.1186/s12967-026-09012-1.

## Background

Effective anticancer immunity correlates with the mobilization of B cells and T cell responses [1]. Endogenous retroviruses (ERVs) are remnants of ancient retroviral infections that are integrated into the germline [2]. In animals, they are effective targets for anti-cancer B and T cell immunity, yet to be translated into clinical trials [3, 4]. The human ERV (HERV) sequences have undergone considerable mutational decay over millions of years [5], with evidence suggestive of negative selection pressure on the two structural proteins envelope (ENV), and group-specific antigen (GAG) [6]. For the past 30 years, evidence has accumulated linking HERVs to cancer pathogenesis [7, 8] and many studies suggest HERVs to be immunologically relevant targets for anti-cancer therapies [9–11].

In previous studies investigating GAG and ENV of Murine Leukemia Virus (MLV), an endogenous γ-retrovirus in mice, unresponsiveness was observed to the DNA vaccines, which could be overcome by exchanging the DNA vectors to adenoviral vectors [12]. Mutation of the immunosuppressive domain (ISD) located in the transmembrane domain of the ENV protein, further augmented the immunogenicity and the anti-cancer efficacy [4]. Together with similar findings using HERV-W/Syncytin-1 ENV protein in mice [13], these studies suggested that ISD mutations in HERVs can provoke an immunological response. The distantly related β-retrovirus HERV-K/HML-2 family is by far the most recently active and structurally intact retrovirus group in the human genome [14] and the most studied HERV in different human cancers as detected by diagnostic biopsies, including with demonstrated spontaneous immunogenicity [7, 15]. The HERV-K/HML-2 family has been reported to be subject to cancer phenotype specific regulation [16] and correlation with treatment sensitivities which might indicate altered expression after initial treatments of cancer patients [17].

In an effort to explore the translational relevance of HERV-K for immunotherapy in cancer patients, we initially confirm the general expression of HERV-K in a vast array of cancers by aligning total RNA reads to 91 full-length or near full-length loci in a large sample of advanced and predominantly heavily pre-treated cancer patients. As the β-retroviral HERV-K ISD peptide does not contain independent immune suppressive activity [18], we screened for and successfully identified an immunostimulatory ISD mutation allowing us to translate the ISD mutation from mouse ERVs to HERV-K. We found that the mutation induced immune stimulatory pathways in primary human dendritic cells and enabled an immunotherapy candidate to expand human CD4^+^ and CD8^+^ T cells with cancer-targeting potential ex vivo. The mutation further safely induces potent B and T cell immunogenicity in mice and non-human primates and anticancer efficacy in a mouse model, suggesting the suitability of HERV-K-ISDmut antigens for clinical translation.

## Methods

### Ethical statement

Human cancer biopsies for assessment of HERV-K expression were obtained for a cohort of 1353 cancer patients referred to the Phase I Unit as part of the Copenhagen Prospective Personalized Oncology study (CoPPO, single-arm open-label study, NCT02290522) [19]. All rodent experiments and procedures were performed according to the national guidelines and experimental protocols approved by National Animal Experiments Inspectorate (Dyreforsøgstilsynet) (license numbers 2016-15-0201-01131, 2019-15-0201-00203 and 2024-15-0201-01692).

Ethical approval for use of cynomolgus macaques was granted by the primate research center at the Bogor Agricultural University IACUC (Animal Care and Use Committee).

### Analysis of human cancer patient HERV-K expression

Human cancer biopsies for assessment of HERV-K expression were obtained for a cohort of 1353 cancer patients referred to the Phase I Unit as part of the Copenhagen Prospective Personalized Oncology study (CoPPO, single-arm open-label study, NCT02290522) [19]. RNA was purified from 18G needle aspirates and total RNAseq was performed using Illumina sequencing. For determining HERV-K expression, RNA reads were aligned using STAR (v.2.5.2b) to a pseudo-genome created using a FASTA file of 91 sequences from the supplementary material of Subramanian et al. [20]. each sequence representing a pseudo-chromosome. To prevent multimapping reads from being counted at more than one locus, STAR was run with --outSAMmultNmax 1, restricting the alignment output to a single best-scoring alignment per read. Primary alignments (SAM secondary alignment flag excluded) were tallied per pseudo-chromosome and normalized to counts per million total input reads. Total HERV-K load was computed by summarizing counts across all 91 pseudochromosomes per sample. We note that this approach resolves each ambiguous read to a single representative sequence and therefore does not distinguish the exact paralog of origin for individual multimapping reads.

### Cells

Cell lines were cultured in cell line-specific media. Human cancer cell lines used were A549 (ATCC, CRM-CCL-185) in Ham’s F-12 (Gibco, 31765035), SK-MES-1 (ATCC, HTB-58) in EMEM (ATCC, 30-2003), NCI-H460 (ATCC, HTB-177), NCI-H1975 (ATCC, CRL-5908) and HCC827 (Gift from Mette Ørskov Agerbæk, UCPH) in RPMI 1640 (Gibco, 61870044), SK-OV-3 (ATCC, HTB-77) in DMEM/F-12 (Gibco, 11330032), and PA-1 (ATCC, CRL-1572) and Ca-ov-3 (ATCC, HTB-75) in DMEM (Gibco, 61965059). Murine cancer cell lines used were Renca (ATCC, CRL-2947), Renca-HKENV (HERV-K ENV expressing Renca cells, kindly provided by Barbara Schnierle, Paul-Ehrlich-Institut, Langen, Germany), CT26 (ATCC, CRL-2638) and CT26-HK (HERV-K GAG and ENV expressing CT26 cells) in RPMI 1640 (Gibco, 61870044). HEK293 and HEK293T cells were cultured in DMEM (Gibco, 61965059). All cell media were supplemented with 10% fetal bovine serum (Merck, F9665-500ML), 1 mM Sodium Pyruvate (Gibco, 11360070) and 1% penicillin-streptomycin (Gibco, 15140122).

### MHC-I epitope prediction

MHC class I CD8 T cell epitopes were predicted with NetMHCpan-4.1, which uses an artificial neural network trained on combined eluted-ligand and binding affinity data, for 139 HERV-K (HML-2) *gag* and *env* open reading frames retrieved from the HERVarium repository (https://zenodo.org/records/19334775) [21]. ORFs were assigned to gag or env by conserved retroviral protein-domain annotation (HMM/GyDB). Binding was predicted for 9-mer peptides against the 20 most common HLA class I alleles: HLA-A01:01, -A02:01, -A03:01, -A11:01, -A23:01, -A24:02, -A26:01, -A32:01, -B07:02, -B08:01, -B15:01, -B18:01, -B35:01, -B40:01, -B44:02, -B44:03, -B51:01, -B57:01, -C05:01 and -C07:02. Peptides were classified as strong binders at an eluted-ligand rank threshold of %Rank_EL < 0.5, and the number of strong binders was summed per provirus across all ORFs and alleles. Sequence similarity of HERV-K ORFs to the HERV-K-ISDmut immunogen was assessed by protein BLAST (blastp) reporting, per ORF, the percent amino acid identity of the best scoring alignment.

### Plasmids and immunization vectors

The HERV-K transgene was designed using codon-optimized HERV-K consensus *gag* and *env* separated by a Glycine/Serine/Glycine (GSG) linker followed by a self-cleaving porcine teschovirus-1 2A peptide (P2A). The sequence was inserted into adenoviral shuttle plasmids and ISD point mutations performed in these. The HERV-K *env* plasmid encoded consensus HERV-K *env* in an adenoviral shuttle plasmid. Adenovirus genomes were generated by bacterial recombination [4, 22]. Codon-optimized mRNA encoding the consensus HERV-K *gag* sequence linked to HERV-K *env* (WT or ISDmut) via a GSG linker followed by a P2A peptide were synthesized and formulated (DSPC, Cholesterol, ALC-0315, ALC-0159 (PEG)) by TebuBio.

HERV-K envelope transmembrane ectodomain proteins (amino acids 495–632), either with the WT or ISDmut (Q525A point mutation) sequence, were manufactured in baculovirus-infected insect cells, as previously described [13].

Gal4-vp16, and pGL4.31 plasmids have been described previously [23].

### HERV-K ENV fusion assay

The cell-cell fusion assay is based on HERV-K ENV effector cell fusion with target cells. For target cells, 8 × 10^5^ HEK293 cells (90–95% confluency) were seeded per well in 6 well plates. For effector cells, 2 × 10^4^ HEK293 cells per well were seeded in poly-D-lysine-coated (20 µg/mL) white 96 well plates (Greiner bio-one). Cells were incubated overnight and transfected the next day using lipofection (Lipofectamine 2000^®^, Thermo Fisher). Target cells were transfected with 1.1 µg pGL4.31 per well. Effector cells were co-transfected with 25 ng of either HERV-K *gag*-P2A-*env* or HERV-K *env* plasmids, and 40 ng gal4-vp16 plasmid per well. Cells were cultured at 5% CO_2_, 95% humidity, and 37 °C for 24 h after transfection. Target cells were detached using Versene (Substrate department, University of Copenhagen, Denmark) and resuspended in antibiotic-free growth medium. Growth medium was removed from the effector cells, and 2 × 10^4^ target cells in 100 µL growth medium were overlaid for each well and co-cultured overnight at 5% CO_2_, 95% humidity, and 37 °C. After co-culture, cells were washed with 100 µL HBSS (Gibco) with 10% FBS and 1% penicillin-streptomycin and luciferase activity was assayed with the Firefly Assay Buffer from Baker et al. [24]., using an EnVision Multilabel Reader (Model 2104, Perkin Elmer).

### HERV-K transfections and NF-κB activity measurement

HEK293T cells (90–95% confluency) were seeded at 35,000 cells/well in poly-D Lysine-coated (20 µg/mL) white 96 well culture plates 24 h prior to transfection. Transfections were performed using the Lipofectamine^®^ 2000 reagent (Invitrogen) with 50 ng/well of the dual luciferase reporter plasmid pNF-κΒ-RE-Ffluc-pSV40-Rluc and concentrations from 0 to 50 ng/well of HERV-K *gag*-P2A-*env* plasmids. Total DNA amount was adjusted with an empty plasmid to 100 ng DNA/well. Cells were cultured at 5% CO_2_, 95% humidity, and 37 °C for 24 h after transfection. Cells were washed twice with 100 µL PBS. For acquiring Firefly luminescence, cells were incubated for 10 min at room temperature in 50 µL of PBS with 50 µL of Dual-Glo^®^ Luciferase Assay Reagent (Promega) per well and luminescence was measured in an EnVision multilabel reader (model 2104, PerkinElmer^®^). Subsequently, 50 µL/well of Dual-Glo^®^ Stop & Glo^®^ Reagent (Promega) was added to the same wells and incubated for 10 min, before acquiring Renilla luminescence. For data analysis, Firefly and Renilla luminescence of HERV-K *env* ISD mutants were normalized as fold change to WT HERV-K *env*. The ratio of NF-κΒ-specific Firefly luminescence over background Renilla luminescence was calculated to account for transfection efficiency and cell viability. Samples were analyzed in triplicate.

### Animals

Wistar outbred female rats (Crl: WI) were purchased from Charles River. CB6F1, C57BL/6 and BALB/c mice were purchased from Envigo (Scandinavia). All studies were performed with female mice except for studies with transgenic mice. Upon arrival, rodents were acclimatized for one week prior to initialization of an experiment.

The in-house transgenic (Tg) mouse model was generated by pronuclear injection of a HERV-K *gag* and *env* transgene (without ISD mutation) under the MMTV promoter into a fertilized oocyte which was then inserted into pseudo-pregnant C57BL/6 mice. The expression of the transgene was regulated under the Tet-On system allowing minimal expression of the transgene. The generated mice were subsequently crossed with WT BALB/c mice to generate Tg CB6F1 mice.

Female cynomolgus macaques (Macaca fascicularis) were about 7 years old with a weight of 2.5–5 kg. Three animals (ID: J041109B, J070510A, J300409B) were selected based on the absence of tuberculosis, simian T-cell leukemia virus and simian retrovirus infections. The selected animals were acclimatized for a month before the start of the trial. Animal handling procedures (blood collection, vaccination) were performed under anesthesia (ketamine and xylazine) by highly trained veterinarians and animal care takers. Animals were housed in groups. They were given an enrichment environment and were fed with monkey chow (Charoen Pokpand, Thailand) twice a day. For each procedure, the animals were monitored for pain, distress, body temperature and weight.

### In vivo immunizations and treatments

Mice were immunized using the vectors described above. Mice were immunized s.c. with a total volume of 30 µL in the footpad or hock [25] or i.m. with a total volume of 100 µL split evenly between the right and left hind leg. Mice immunized with proteins were immunized s.c. with 5 µg protein formulated in a total volume of 100 µL of AddaVax (Invitrogen) and PBS mixed 1:1. Rats were immunized s.c. in the hock with a total volume of 100 µL. Cynomolgus macaques were immunized i.m. with a total volume of 1 mL. Further information on immunization routes, dosage and regimen can be found in the figure legends. Anti-PD-1 and isotype control antibody were administered intraperitoneally (i.p.).

### HERV-K peptides

Peptide pools were designed to cover the HERV-K GAG and ENV consensus sequences [26]. These consisted of 16-mer peptides overlapping by 11 amino acids. The peptides were divided into pool 1 (HERV-K GAG sequence 1–62 aa, Karebay), pool 2 (HERV-K GAG sequence 63–133 aa, Karebay), pool 3 (HERV-K ENV SU sequence 134–225 aa, Karebay) and pool 4 (HERV-K ENV TM sequence 226–271 aa, Karebay). Peptide pools were produced and lyophilized by Karebay Biochem (USA), purified by HPLC with a purity > 80%. During the production, the following four peptides were of low purity: MLLAALMIVSMVVSLP (50%), EEEGMMINISIGYRYP (70%), LVWEECVANSAVILQN (50%), and AVILQNNEFGTIIDWA (60%); and 5 could not be produced: FIFTLIAVIMGLIAVT, GLIAVTATAAVAGVAL, KTIGSTTIINLILILV, TTIINLILILVCLFCL, LILILVCLFCLLLVCR, and were therefore not present in peptide pools 1–4. The single ENV peptide 18 with the sequence TYHMVSGMSL was produced with a purity of > 80% (Shafer-N).

### Murine cancer models

Renca-HKENV mouse renal carcinoma tumor cells engineered to express HERV-K ENV were cultured as stated above. HERV-K expression was determined by fluorophore staining and flow cytometry as described below. Cells were harvested at 70–80% confluency using pre-warmed trypsin (25300054, Gibco). Following detachment, cells were washed twice with cold PBS. Tumor cell suspension was administered i.v. via the lateral tail vein at 5 × 10^5^ cells in 100 µL. Post-injection, cell viability and concentration were reassessed to confirm consistency. Mice were randomized post tumor cell administration except when immunized prophylactically. Tumor size was measured in length and width 2–3 times weekly, and the tumor volume was calculated as: length × width^2^ × 0.5236 [27]. Quantification of tumor nodules post Renca-HKENV tumor challenge was done on whole lungs fixed in 4% paraformaldehyde (PFA) and stored in 70% ethanol. Samples were randomized prior to assessment. Tumor burden was evaluated by visually inspecting and counting all surface tumor nodules. For survival, mice were monitored 3–5 times per week following intervention to assess health status and survival. In the Renca-HKENV survival study, the investigator monitoring survival was blinded to treatment allocation. Euthanasia was performed upon the emergence of humane endpoint criteria.

### Healthy human donor PBMCs

Buffy-coats from anonymous human donors were acquired from the blood bank at Rigshospitalet (Copenhagen, Denmark). PBMCs were isolated and cryopreserved. Monocytes were isolated, cultured, differentiated and matured as previously described [28]. Where indicated in the figure legends, cells were maintained in an immature state. On day − 1 (7 days post monocyte isolation), cells were transduced with a multiplicity of infection (MOI) of 50 using Ad19a or Ad5F35 vectors. Differentiated and matured monocyte-derived DCs (moDCs) were co-cultured with autologous PBMCs at a ratio of 1:10 using 1 × 10^5^ moDCs and 1 × 10^6^ PBMCs per well in 48 well plates (61965059, Thermo Fisher Scientific). Cultures were maintained in X-Vivo 15 (LZ-02-060Q, Lonza) supplemented with 10% human serum (H5667, Sigma-Aldrich). Co-culture started on day 0, 2, 7 and 9. Between day 2 and 7, media was changed and supplemented with IL-2 (130-097-746, Miltenyi Biotec) at 100 IU/mL every 2–3 days. Cells were passaged when the phenol red turned orange in less than 2 days. After 4 h of stimulation with 2 µg/mL of a peptide pool containing HERV-K peptide pools 1–4, IFN-γ producing PBMCs were sorted and subsequently rapidly expanded as previously described [28].

### Library preparation and sequencing of human moDCs

Samples for RNA isolation and sequencing were collected from immature human moDCs at 48 h after transduction. Libraries were prepared using Illumina Stranded Total RNA Prep with Ribo-Zero Plus protocol and sequencing was performed on NovaSeq X Plus − 10B flow cell. Bulk RNA sequencing was carried out by the Department of Genomic Medicine at Rigshospitalet, Denmark. Roughly 300 million paired reads were obtained per sample.

### Differential gene expression and pathway enrichment analysis

Bulk RNA sequencing was performed on monocyte-derived dendritic cells from 6 healthy donors, each transduced with a wild type (WT) or a modified (ISD) HERV-K-encoding vector. Transcriptome sequencing RIN scores were similar between WT and ISDmut conditions and all samples had above 95 million reads. Raw paired-end FASTQ files were mapped to standard human reference (Hg38) using Spliced Transcripts Alignment to Reference (STAR) aligner. Merged RNA-seq raw gene-level count data were imported into the statistical software R.

Counts were imported into edgeR as a DGEList, and a design matrix accounting for patient identity and vector condition (~ patient + vector) was used to remove genes with insufficient expression via filterByExpr. Filtered counts were then analyzed with DESeq2, fitting a negative binomial generalized linear model with the same paired design (~ patient + vector) to account for the within-patient structure of the experiment. One donor (D2) contributed only an ISD sample due to poor data quality from sequencing of the WT counterpart. D2 is retained in the model as it provides information for estimating the donor-level baseline effect, though contributing with no within-donor ISD vs. WT contrast. Differential expression between vector conditions were assessed using the Wald test, with the contrast set to ISD versus WT. Fold-change shrinkage was applied for ranking (ashr) and Benjamini-Hochberg adjusted p-values used for significance testing (*P* < 0.05).

Hallmark H collection from MSigDB were retrieved and gene set enrichment analysis (GSEA) was performed fgseaMultilevel, with genes pre-ranked by shrunk log2 fold-change. Custom thematic pathway collections were assembled from the Reactome database (MSigDB C2:CP: REACTOME subcollection) by selecting gene sets corresponding to senescence, antigen presentation and cell death. Gene IDs were obtained for significant genes by comparison to gene names with GRCh38. Visualizations were done using ggplot2 and pheatmap.

Sample-level quality assessment was performed on the filtered count matrix after variance-stabilising transformation (DESeq2 vst) with principal component analysis on the 1000 most variable genes while removing inter-donor variation (limma::removeBatchEffect). The analysis was repeated on the five donors contributing complete WT/ISDmut pairs. Principal components were tested for association with donor, vector and technical covariates (library size, RNA integrity number, RNA input concentration).

### HLA typing

DNA was extracted from donor PBMCs and cancer cell lines using the DNeasy Blood & Tissue Kit (69504, Qiagen) according to the manufacturer’s instructions. HLA typing was then performed by DKMS Life Science Lab (Germany).

### Staining cancer cells and moDCs for flow cytometry

For analysis of surface HERV-K expression by flow cytometry, cells were incubated with Fixable Viability Dye eFluor780 (13539140, Invitrogen) 1:1000 for 30 min at 4 °C in the dark and subsequently blocked with Human TruStain FcX (422302, BioLegend) 1:20 for 10 min at 4 °C in the dark. Surface staining was performed using an anti-HERV-K ENV antibody (HERM-1811-5, Austral Biologicals) at 1:100 conjugated to AF647 using the Alexa Fluor 647 Antibody Labeling Kit (A20186, Thermo Scientific) or a matched isotype control (Mouse IgG2a kappa, 16557070, Invitrogen) conjugated to AF647 1:20 in FACS buffer (PBS containing 1% BSA and 0.1% NaN3) for 30 min at 4 °C in the dark. Cells were washed and fixed with 1% PFA for 20 min at 4 °C in the dark. After staining, cells were washed and resuspended in FACS buffer for analysis by flow cytometry. For the Renca/Renca-HKENV and CT26/CT26-HK specifically, cells were first stained with HERM1811-5 (Ango) conjugated to AF647 at 1:100 for 30 min, followed by incubation with Fixable Viability Dye eFluor780 (13539140, Invitrogen) 1:1000 for 20–30 min, and fixed with 1% PFA for 15 min. All incubations were performed in the dark at 4 °C.

For the staining of HEK293 cells transfected with either a plasmid encoding HERV-K *gag*-P2A-*env* or *gag*-P2A-*envISDmut*, cells were blocked with goat serum for 10 min at room temperature and then incubated with a primary antibody at 10 µg/mL (Kenv-1, Kenv-2, Kenv-4, Kenv-6, Kenv-7, Kenv-14; Erica Ollmann Saphire, La Jolla Institute for Immunology, La Jolla, USA; HERM-1811-5, Austral Biologicals) for 30 min at 4 °C in the dark. Cells were washed and incubated with either Goat anti-mouse IgG conjugated to APC (405308, Nordic Biosite) or Goat anti-Human IgG conjugated to Alexa Fluor 647 (A55749, Thermo Fisher) for 20 min at 4 °C in the dark. Cells were washed and incubated with the viability dye eFluor450 (65-0863-14, eBioscience) 1:1000 for 20–30 min at 4 °C in the dark. Cells were subsequently washed, fixed, and washed as above.

To generate antibodies for IgG-specific HERV-K detection, rats were immunized with a prime boost regime of Ad19a/Ad5F35-HERV-K-ISDmut, while control sera were obtained from animals receiving empty Ad19a/Ad5F35 vectors. IgG was subsequently purified from serum by Protein G affinity chromatography using Gammabind Plus Sepharose (GE17-0886-01, Sigma-Aldrich), followed by buffer exchange and concentration. Purified IgG was then conjugated to Alexa Fluor 647 using the Alexa Fluor 647 Antibody Labelling Kit (A20186, Thermo Scientific). For HERV-K detection using serum of HERV-K-immunized rats, cancer cells were stained with Fixable Viability Dye eFluor780 (13539140, Invitrogen) 1:1000, fixed in 1% paraformaldehyde, and permeabilized with 0.5% saponin. Cells were then blocked with serum from control-immunized rats (1:10) and stained with AF647-conjugated HERV-K IgG or AF647-conjugated control IgG (1:50 in 0.5% saponin buffer). After washing, cells were resuspended in FACS buffer for analysis by flow cytometry.

For analysis of antibody responses induced by protein immunization, the antibody titers specific for ENV domain of HERV-K were measured by serum binding to CT26 cells expressing HERV-K GAG and ENV (CT26-HK). Briefly, on the day of the experiment, cells were detached with 0,05% trypsin-EDTA (1x) and seeded at a concentration of 250.000 cells per well in 96-well Clear Round Bottom TC-treated Micro plate (Corning). Serum obtained from mouse blood was diluted 1:50 in FACS buffer (containing 1% BSA, 0.1% NaN_3_). Cells were washed twice in FACS buffer and 50 µL of prepared serum dilutions were added per well. Cells were incubated with primary antibodies for 60 min at 4 °C and washed twice in FACS buffer. Cells were then incubated with PE-conjugated secondary antibody (1:100 Goat anti-mouse IgG, Biolegend) and viability dye (1:1000, eFluor780, Invitrogen) for 30 min at 4 °C, followed by washing and fixation of cells in 1% PFA 15 min at 4 °C. After washing, cells were resuspended in FACS buffer for analysis by flow cytometry.

### PBMC stimulation and intracellular staining for flow cytometry

Human PBMCs were restimulated and assessed by surface and intracellular staining (ICS) as previously described [28] using peptide pool 1 (HERV-K GAG), pool 2 (HERV-K GAG), pool 3 (HERV-K ENV) and pool 4 (HERV-K ENV) at 1 µg/mL for stimulation. For stimulation with the HLA matched cancer cell line HCC827, cancer cells were seeded into a round bottom 96-well plate at 5 × 10^4^ cells per well together with either HLA-A, B and C blocking antibody (311402, BioLegend) or an isotype control (401202, BioLegend) at 20 µg/mL for 1 h at 37 °C and 5% CO_2_. Cancer cells were then co-cultured with 2.5 × 10^5^ non-expanded PBMCs or HERV-K stimulated PBMCs per well (1:5 ratio of cancer cell to PBMC) for 5 hours at 37 °C in 5% CO_2_.

For murine PBMCs, spleens were mashed and incubated with relevant peptides, which was either the above-mentioned HERV-K peptide pools 1–4 or the specific single (peptide 18, ENV, TYHMVSGMSL), to stimulate cytokine production for 5 h at 37 °C, 5% CO_2_ in the presence of 4 µM monensin. For surface staining, cells were stained with fluorescent-labelled antibodies directed against: CD8 (BV421, 126629, BioLegend); CD4 (PE-Cy7, 552775, BD Biosciences); B220 (PerCP-Cy5.5, 552771, BD Biosciences); CD44 (FITC, 553133, BD Biosciences) in FACS buffer for 20 min at 4 °C in the dark. Cells were washed and fixed with 1% PFA for 20 min at 4 °C in the dark. Following washing, cells were stained with intracellular antibodies directed against IFN-γ (APC,554413, BD Biosciences) and TNF-α (PE, 554419, BD Biosciences) in 0.5% Saponin for 20 min at 4 °C in the dark. After washing, cells were resuspended in FACS buffer for analysis by flow cytometry. For tetramer staining, whole blood (collected in EDTA tubes) or splenocytes were subjected to red blood cell lysis with 1X RBC Lysis Buffer (00-4333-57, Invitrogen) for 4–5 min at room temperature. Mouse PBMCs and splenocytes without red blood cells were first incubated with the relevant tetramer. Typically, 0.08 µg to 0.25 µg of the relevant tetramers in FACS buffer with 50 nM of Dasatinib were added to 400 000 cells at 37 °C, 5% CO_2_ for 15 min (tetramer mix). Surface antibodies (CD8b-BV510; CD4-PE-Cy7; B220-PerCP-Cy5.5; CD44-FITC) and viability dye (Efluor780) in FACS Buffer with 50nM of Dasatinib (CDS023389, Sigma-Aldrich) were then added and incubated for 20 min at 4 °C. Cells were washed and fixed with 1% PFA for 20 min at 4 °C. After washing, cells were resuspended in FACS buffer for analysis by flow cytometry.

### Flow cytometry

Fixed samples from CT26 cell binding assay, tetramer staining, surface staining, or ICS were run on either BD LSR Fortessa 3, 5, or X20 flow cytometers (BD biosciences) and analyzed using FlowJo software V10.7.1.

### Enzyme linked immunosorbent assay (ELISA)

96-well flat-bottom plates were coated with either proteins or antigen-expressing cells. HERV-K ENV SU (ERVK-6 ENV SU manufactured by Kactus or consensus HERV-K ENV SU produced by the Discovery Group, Centre for Medical Parasitology, University of Copenhagen, Denmark) were used at 2 µg/mL in PBS and left overnight at 4 °C. The next day, the plates were covered with blocking buffer (20.7 g/L of NaCl, 5 g/L of BSA, and 0.05% (v/v) of Tween-20 in PBS (-Mg and Ca), pH 7.2) for 1 h at room temperature (RT) to minimize non-specific protein binding. In the cell-based ELISA, CT26-HK or CT26-WT cells were plated for 2 h at 37 °C with 5% CO_2_. The cells were then fixed with 4% PFA. The plates were blocked overnight at 4 °C as above. Plates were incubated with serum samples diluted as indicated in the figures for 1 h. Secondary horseradish peroxidase (HRP)-conjugated polyclonal antibodies were then added: either a goat anti-mouse IgG (P0260, Dako) or a rabbit anti-monkey (A2054, Sigma-Aldrich) diluted at 1:2000 in blocking buffer. Plates were incubated for 1 h at RT. Plates were washed between each step. Bound antibodies were detected using TMB PLUS2 (4395A, Kem-En-Tec Diagnostics), and the colorimetric reaction was stopped after approximately 8 min by adding 0.2 M H_2_SO_4_. The absorbance at 450 and 650 nm was subsequently assessed with an ELISA plate reader (SpectraMax or VersaMax, Molecular Devices, or Agilent BioTek Epoch 2). The reading at 650 nm was not performed in some experiments.

### Transmission electron microscopy (TEM)

Human moDCs were generated as described above and once matured they were seeded in 24 well plates on Thermanox coverslips (150067, Thermo Fisher) coated with Poly-L-Lysine. Cells were transduced with either HERV-K-WT, HERV-K-ISDmut or Empty Ad19a vector (25 MOI). 48 h post transduction; cells were fixed in 2% glutaraldehyde (0.05 M sodium phosphate buffer, pH 7.2) and postfixed in 1% OsO_4_ (0.12 M sodium phosphate buffer, pH 7.2) for 2 h. Samples were dehydrated through graded series of ethanol, transferred to propylene oxide, and embedded in Epon. Sectioning and examination were performed as previously described [4].

### Western blot (WB)

For analysis of HERV-K expression, protein was isolated by lysing cell pellets in RIPA buffer (89901, Thermo Scientific) supplemented with Protease/Phosphatase Inhibitor (78440, Thermo Scientific) and EDTA (78440, Thermo Scientific). Protein concentrations were determined by a bicinchoninic acid (BCA) protein assay (17888228, Thermo Scientific). Reduced proteins (20 µg) were resolved by sodium dodecyl sulfate polyacrylamide gel electrophoresis (SDS-PAGE) and transferred to nitrocellulose membranes by electroblotting. Membranes were stained with Ponceau S Staining Solution (A40000278, Thermo Scientific) to confirm transfer, washed, and then blocked in 5% milk in TBS-T buffer. Primary staining was performed overnight at 4 °C using rat serum diluted in 3% milk/TBS-T. The serum was obtained from rats immunized with Ad19a/Ad5F35-HERV-K-ISDmut or from control rats immunized with empty Ad19a/Ad5F35 vectors. After incubation with HRP-conjugated anti-rat IgG (31470, Invitrogen) diluted in 3% milk/TBS-T, detection was performed using KPL Lumiglo Solution (KPLI5430-0040, SeraCare), followed by image acquisition on the iBright CL1500 (Invitrogen). For β-actin detection, membranes were stripped and staining performed using an anti-β-actin antibody (115777, Abcam), followed by HRP-conjugated anti-rabbit IgG (P0448, Dako). Imaging was repeated using the same system.

### Statistical analyses

Individual values are presented as means with error bars represented as SEM. Statistical analyses were performed using R version 4.4.3 (2025-02-28) or GraphPad Prism versions 9 and 10 for Windows (GraphPad Software) and as described in each figure legend.

## Results

### HERV-K is broadly expressed in human cancers

**Fig. 1:**
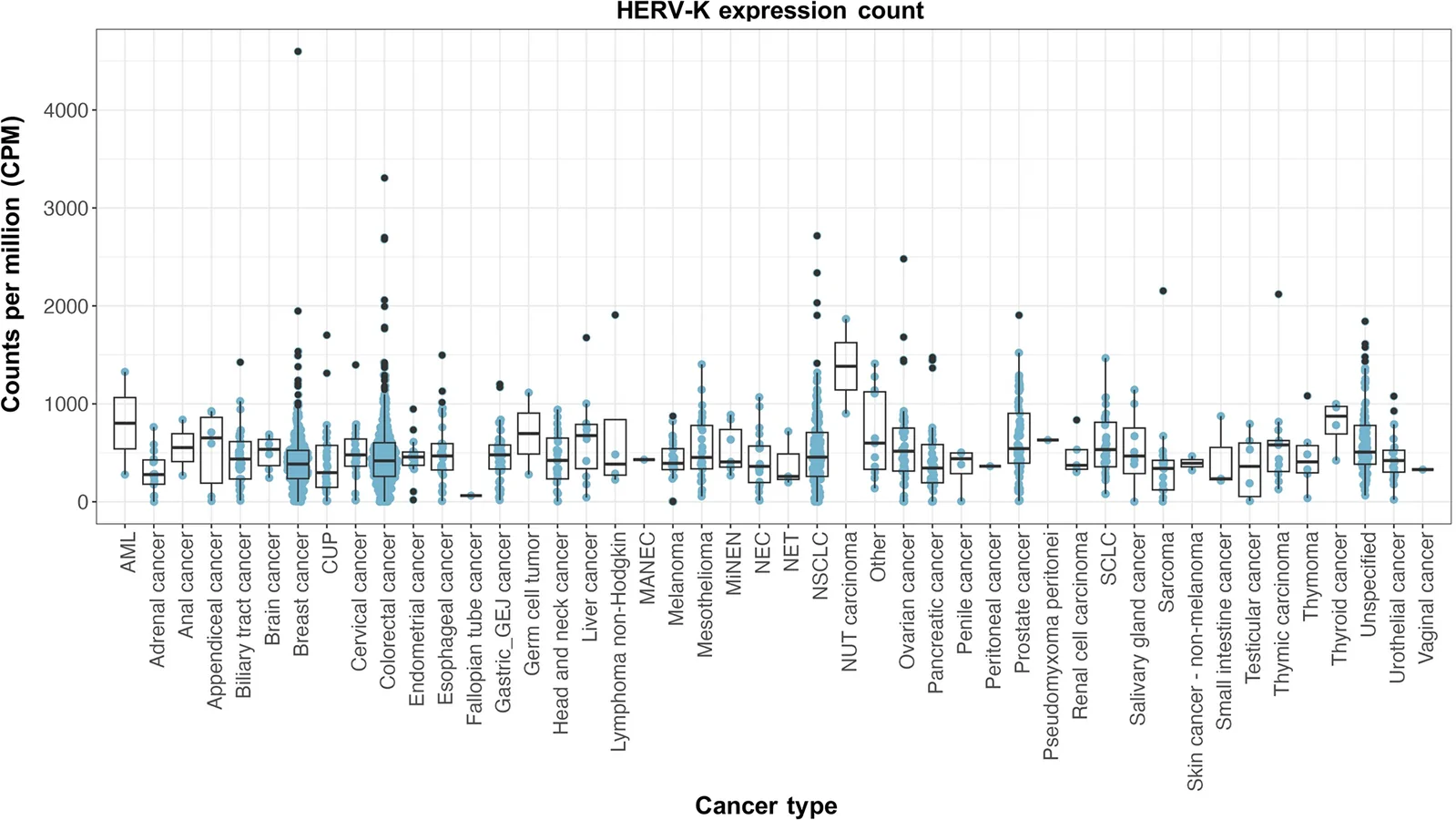
HERV-K is expressed in cancer patients. Normalised HERV-K transcriptional activity across late-stage solid tumor samples from 1353 patients sequenced as part of the Copenhagen Prospective Personalised Oncology Study (CoPPO). Mapping RNA-seq data to 91 near full-length HERV-K sequences in the human genome reveals high-level HERV-K expression (> 100 reads/million) across the majority of the 43 examined cancer types, cancers of unknown primary (CUP), and unclassified cancers (“Other” and “Unspecified”). Cohort composition is listed in Supplementary Figure S1

While protein-coding HERV-K has been characterized in several solid tumor types and found to be expressed at levels beyond healthy control tissues [7–10, 15–17, 29, 30], the breadth of antigen expression has not been systematically assessed, which raises a question of whether possible publication biases underlie the selected cancer settings where protein expression has been detected (reviewed in [7]). Additionally, it has been reported that lung squamous cell carcinomas can undergo a shift from HERV-H expression in early disease to a HERV-K-associated transcriptomic profile in later and treatment resistant cancers [16]. To explore the breadth and persistence of HERV-K expression, we assessed HERV-K expression in 1353 patients of advanced, mostly pre-treated cancer, for whom total RNA-seq data were available from biopsies collected after referral to a phase 1 trial unit. The dataset does not include matched normal tissue controls. After aligning the RNA-seq data to 91 near full length HERV-K/HML-2 loci in the human genome [20], HERV-K was found in nearly all analyzed cancer types, with the majority of the 1353 patients having more than 100 HML-2 reads per million total reads (Fig. 1).

### Identification of a proinflammatory mutation within the HERV-K ISD

**Fig. 2:**
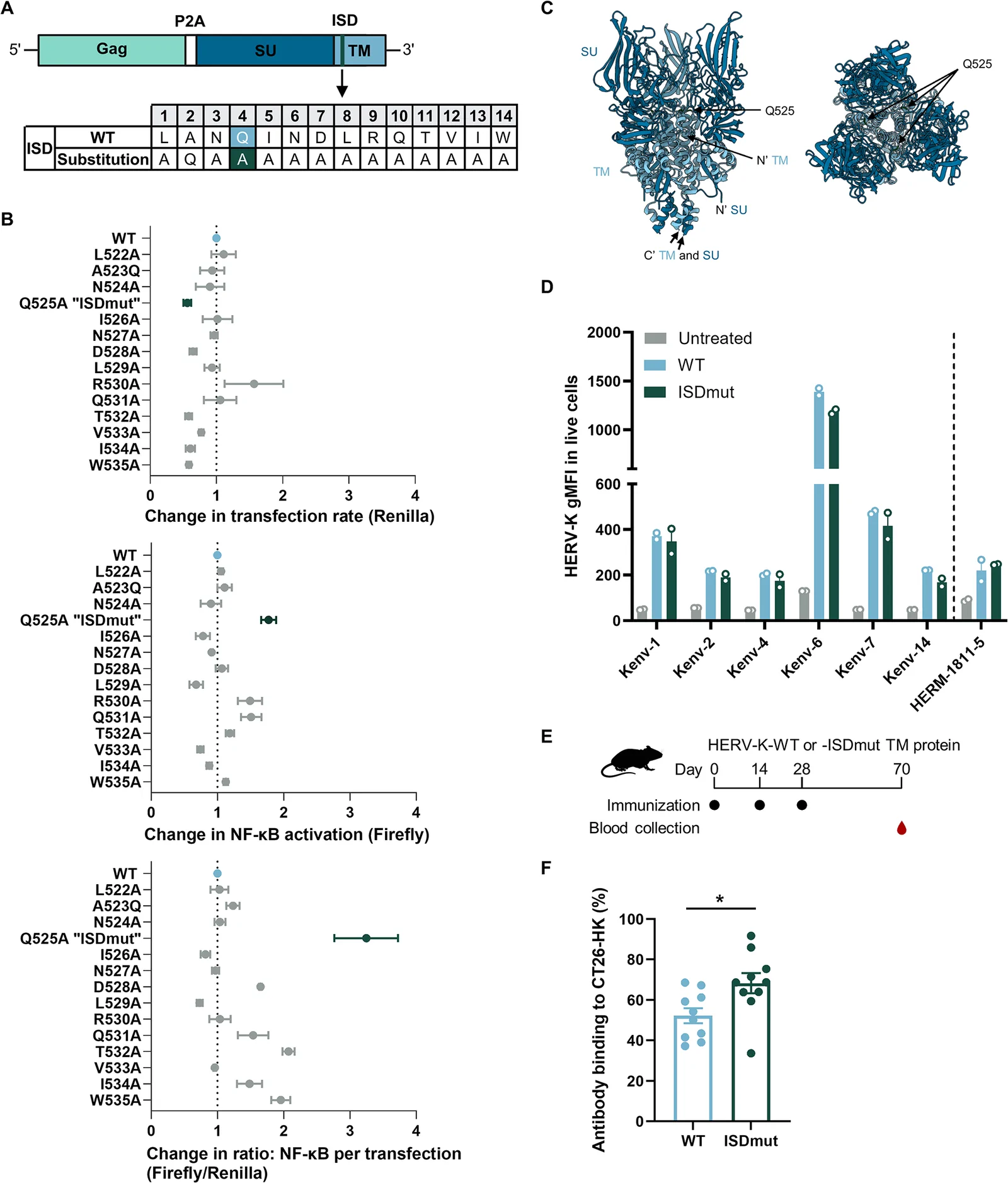
Mutation of the HERV-K ENV ISD leads to increased NF-κΒ activation and higher HERV-K antibody binding in mice. **A**, Graphic design of the antigen cassette, encoding HERV-K consensus *gag* and *env*. The ISD is indicated in the image, and the ISD sequence is listed in the table below. The individual amino acids in the ISD were mutated as indicated in the table. The Q525A amino acid substitution is termed ISDmut in this publication. **B**, HEK293T cells were transfected with a HERV-K *gag-*P2A-*env* encoding plasmid and tested against 14 different amino acid substitutions in the ISD. All plots depict the relative fold change in luminescence to non-mutated conditions (WT). Upper graph shows transfection rate, while middle graph shows NF-κΒ activation. Lowest graph shows the NF-κΒ activation per transfected live cell. All measurements were performed in triplicates; error bars represent SEM. **C**, Side- and top view of HERV-K ENV prefusion structure (PDB:9NND). The residue Q525 of the ISD is shown in dark green (structure modified with UCSF ChimeraX [35]). **D**, Flow-cytometric characterization of HERV-K ENV surface expression on untreated HEK293 cells, transfected with HERV-K-WT or transfected with HERV-K-ISDmut using monoclonal antibodies specific for either linear epitopes located in the transmembrane unit (Kenv-1, Kenv-2, Kenv-7, HERM-1811-5), or surface unit (Kenv-14), or monoclonal antibodies specific for a conformational epitope in the transmembrane unit (postfusion, Kenv-4) or the surface unit (prefusion, Kenv-6). Bars represent gMFI values for each condition. **E**, Protocol outline (created with BioRender.com). BALB/c mice (*n* = 10) were immunized with the HERV-K ENV TM ectodomain (amino acids 495–632), either with wild-type ISD (WT) or with the ISD amino acid substitution Q525A (ISDmut). Mice were immunized subcutaneously (s.c.) in the right flank. **F**, Analysis of serum collected as outlined in **E**. The graph shows flow-cytometric analysis of antibodies binding to HERV-K GAG and ENV expressing CT26-HK cells. *P < 0.05 using a Mann Whitney *U* test

To identify immunogenic variants of the putative ISD in the HERV-K ENV, we encoded the consensus sequences of the HERV-K/HML-2 GAG polyprotein and ENV protein [26] with the two antigens separated by a self-cleavable peptide (P2A) to allow equimolar expression of GAG and ENV as was done for previous ISD mutated ERV immunotherapies [4, 13]. Use of a consensus sequence was selected due to its close homology to intact extant HERV-K insertions; moreover, the vast majority of predicted epitopes in the HERV-K consensus sequence were present in the more intact and recently integrated HERV-K loci containing full-length HERV-K GAG and ENV antigens annotated human loci (Supplementary figure S2) [4, 13]. The construct co-expressing HERV-K GAG and ENV (termed HERV-K “wild-type” (HERV-K-WT)), was found to abrogate the fusion activity of HERV-K ENV (Supplementary figure S3), a finding relevant for the safety profile of the construct. We then systematically substituted each residue in the previously proposed HERV-K ISD [31] (Fig. 2A) and measured NF-κΒ activation in HEK293T cells upon transfection with plasmids encoding the different ISD variants. Interestingly, 6 amino acid substitutions were found to increase the NF-κΒ signal, with the Q525A substitution inducing the highest activation compared to HERV-K-WT (Fig. 2B). Based on recent structural studies, Q525 is located within a loop region in the prefusion conformation of the HERV-K ENV [32, 33] (Fig. 2C) and is located within a “stutter” motif (8 residues) in the heptad-repeat region, a feature that may introduce flexibility within the helices [34]. AlphaFold structural modelling of the Q525A substitution predicted no significant difference in local protein structure compared to the WT Q525 sequence. However, the amino acid substitution from a polar (Q) to a non-polar (A) amino acid could potentially disrupt both intra-subunit hydrogen bonding (helix-loop stabilization) and inter-subunit interactions at the protein interface. To assess the conformation of the HERV-K-WT and the HERV-ISDmut ENVs we used a set of monoclonal antibodies previously developed to characterize the HERV-K ENV structure [33]. The highest binding to ENV on either construct was observed for the Kenv-6 antibody that is specific for a pre-fusion conformational epitope present on the surface unit, indicating that the Q525A mutation in HERV-K-ISDmut does not have a deleterious impact on the conformation of the ENV protein (Fig. 2D). Immunization of BALB/c mice with recombinant HERV-K ENV WT and ISDmut transmembrane domain proteins (Fig. 2E) demonstrated improved ability to induce antibodies towards cells expressing HERV-K-WT on the cell surface (Fig. 2F).

### HERV-K-ISDmut increases antigen presentation and transcriptomic reprogramming in human monocyte-derived DCs

To generate an immunotherapy for further testing, we encoded HERV-K *gag* and *env*, separated with P2A, with (HERV-K-ISDmut) or without the Q525A mutation (HERV-K-WT), in the rare human adenovirus type 19a/64 (Ad19a) [36], and the pre-existing immunity evading type 5F35 (Ad5F35) [37] vectors.

**Fig. 3:**
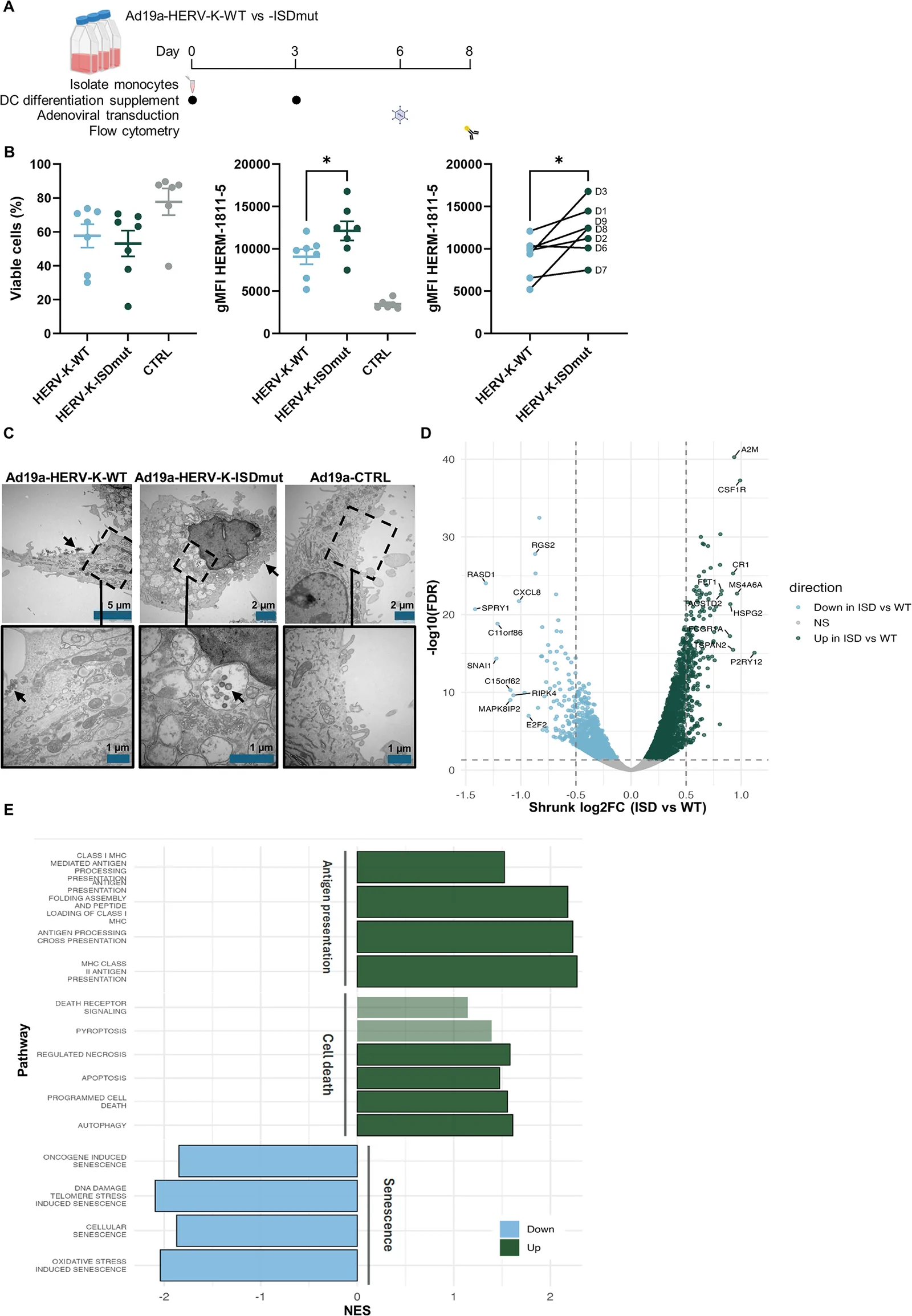
Transduction of human moDCs with HERV-K-ISDmut leads to increased HERV-K ENV expression, formation of VLPs, and activation of distinct cellular pathways compared with HERV-K-WT transduction. **A**, Protocol outline (created with BioRender.com). Monocytes from healthy human donors (*n* = 9) were isolated, treated with DC differentiation supplements at day 0 and 3, transduced with Ad19a-HERV-K-WT, Ad19a-HERV-K-ISDmut, or Ad19a-CTRL (empty vector) on day 6 and then analyzed by flow cytometry on day 8 (48 h post transduction). **B**, Flow-cytometric analysis of human moDCs generated as outlined in **A**. Viability shown as frequency of total cells and HERV-K ENV expression on the surface of live cells shown as geometric mean fluorescence intensity (gMFI) (see gating in Supplementary Fig. S7). Middle and right panels show the same data. Donors 4 and 5 had < 2% of cells on FSC and SCC and were excluded. Error bars represent SEM. **C**, Transmission electron microscopy (TEM) pictures representative of human moDCs (*n* = 1 healthy human donor 15) 48 h post transduction with either Ad19a-HERV-K-WT, Ad19a-HERV-K-ISDmut or Ad19a-CTRL. Images shown at two magnifications, lower (top) and higher (bottom). Virus-like particles are indicated with arrows. **D**, Volcano plot indicating differences in gene expression from human moDCs (*n* = 6) 48 h post transduction with either Ad19a-HERV-K-WT or Ad19a-HERV-K-ISDmut. A total of 23,922 genes were analyzed, with 5503 genes found to be significantly increased or decreased in expression. **E**, Reactome panel analysis from transcriptome sequencing of human moDCs (*n* = 6 healthy donors) transduced with either Ad19a-HERV-K-WT or Ad19a-HERV-K-ISDmut. (**D** and **E**) Donors in this analysis were D2, D4, D5, D6, D7, and D8. One donor (D2), transduced with Ad19a-HERV-K-WT, was excluded due to poor data quality from sequencing. *P < 0.05 by Wilcoxon matched-pairs signed rank test

We investigated the HERV-K transgene expression and its effects over time in human healthy donor monocyte-derived dendritic cells (moDCs) by transducing moDCs with Ad19a encoding either HERV-K-WT, HERV-K-ISDmut, or a control vector that did not encode any transgene (Ad19a-CTRL) (Supplementary Fig. S4). 48 h post-transduction was selected as a compromise between transgene expression and viability to characterize moDCs from 7 donors in a parallel study (Fig. 3A). Across donors, Ad19a-HERV-K-ISDmut induced a significant increase in HERV-K ENV transgene expression compared to Ad19a-HERV-K-WT (Fig. 3B). As observed previously, moDC viability was reduced following transduction with both HERV-K-encoding vectors compared to cells transduced with the control vector (Ad19a-CTRL). To explore the causes of decreased viability we performed transmission electron microscopy (TEM) imaging of transduced moDCs. This revealed formation of VLPs budding off from the cell surface and accumulation of VLPs within intracellular vesicles (Fig. 3C). Both Ad19a-HERV-K-WT and Ad19a-HERV-K-ISDmut transduced human DCs showed signs of extensive cellular disruption with accumulation of intracellular aggregates and prominent vacuolization as compared to Ad19a-CTRL transduced cells.

To explore possible transcriptomic changes induced by HERV-K-ISDmut, we next performed whole transcriptome sequencing in transduced immature moDCs at 48 h post transduction. Material from 6 donors, either transduced with Ad19a-HERV-K-WT or Ad19a-HERV-K-ISDmut was analyzed. Dramatic transcriptional changes were observed from the ISD mutation, with significant differences in approximately 5503 genes, 3479 of which showed increased expression in ISDmut compared to WT (Fig. 3D). To address this, we analyzed specifically the reactome panels with a focus on senescence associated pathways [38], cell death pathways [39] and antigen presentation pathways. The analysis indicated that the ISDmut led to downregulation of senescence pathways as compared to HERV-K-WT, while simultaneously upregulating cell death and antigen presentation pathways (Fig. 3E). Notably, quantitation of transcripts mapping to the codon-optimized HERV-K transgenes demonstrated higher mRNA expression of the HERV-K transgene in HERV-K-WT transduced cells (Supplementary Fig. S5). Importantly, these differences were not due to differences in quality as evidenced by similar yields and quality of the extracted RNA and clear segregation of the two groups principally mediated by the applied vector (WT/ISDmut) on the principal component analysis (Supplementary Fig. S6). This indicated that the observed increases in protein expression and trend towards increased cytotoxicity with HERV-K-ISDmut were not due to differences in transduction level or promoter transactivation.

### HERV-K-ISDmut transduced moDCs expand human HERV-K specific T cells

**Fig. 4:**
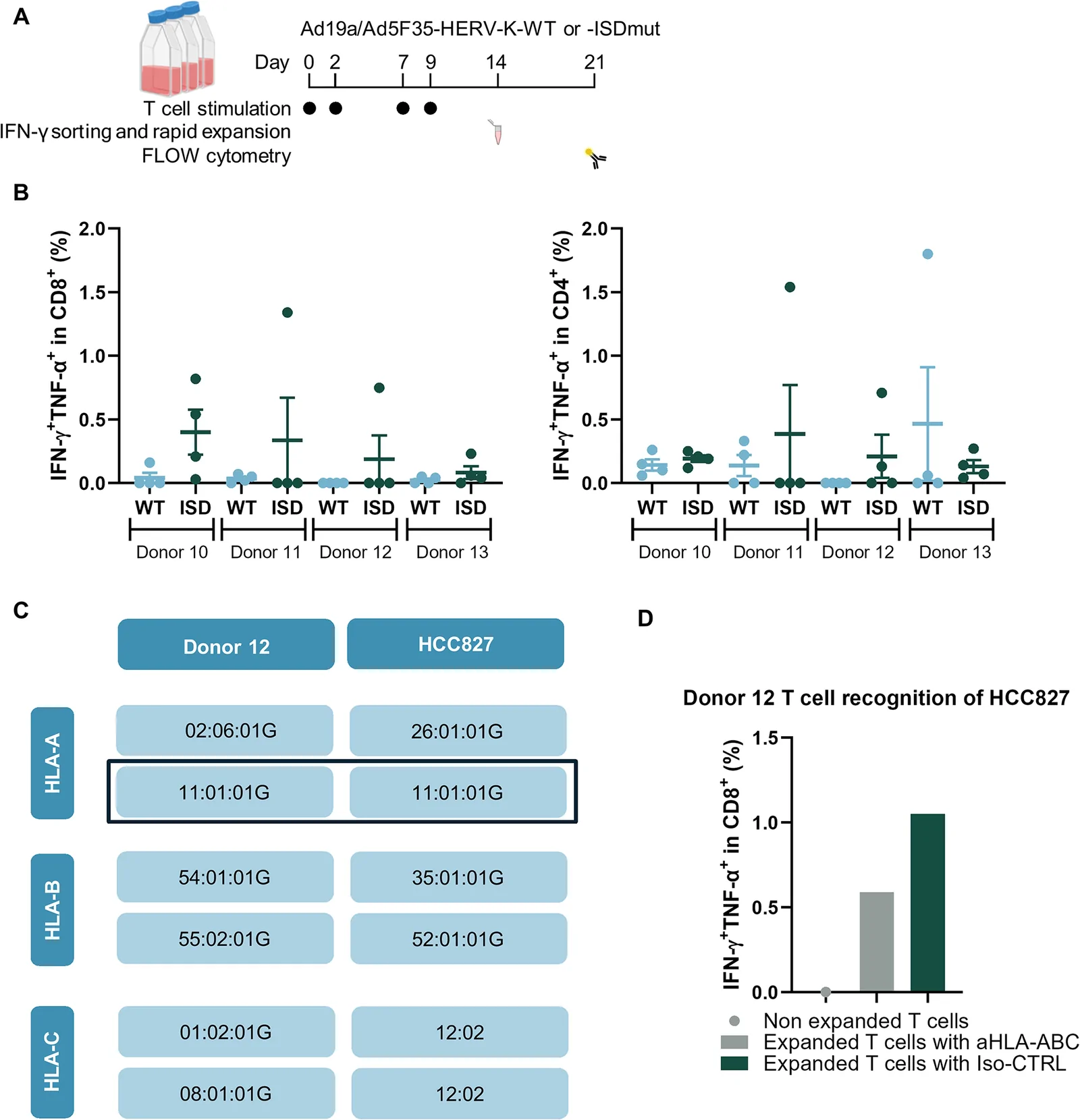
Ad19a-HERV-K stimulation of human PBMCs ex vivo expands human HERV-K specific T cells. **A**, Protocol outline (created with BioRender.com). PBMCs from healthy human donors (*n* = 4). Each donor was stimulated in parallel with autologous mature moDCs transduced with adenoviral vectors Ad19a and Ad5F35 encoding either HERV-K-WT, HERV-K-ISDmut or empty control for 14 days and sorted by IFN-γ capture. Full protocol in materials and methods and Supplementary Fig. S8. **B**, Flow-cytometric analysis of the T cells generated as outlined in **A**. Responses of live, CD8^+^ (left panel) or CD4^+^ (right panel) T cells to HERV-K peptide pools, measured as the frequency of IFN-γ^+^, TNF-α^+^ double-positive cells. Gating strategy in Supplementary Fig. S10. Each dot represents re-stimulation with one individual HERV-K peptide pool (pools 1–4). See Supplementary Fig. S9 for full analysis. **C**, HLA matching of donor 12 and the HCC827 cancer cell line. **D**, Flow-cytometric analysis of responses of live CD3^+^ and CD8^+^ T cells from donor 12 toward HLA-A matched cancer cell line HCC827 as the frequency of IFN-γ^+^, TNF-α^+^ double positive cells. An additional control was added to show the difference in activation with and without the HLA molecules blocked using an HLA-A, B, and C blocking antibody (aHLA-ABC)

We next assessed immunogenicity directly by comparing the capacity of Ad19a-HERV-K-WT and Ad19a-HERV-K-ISDmut transduced moDCs to drive T cell expansion from 4 healthy donor PBMC samples ex vivo. We used an assay previously developed to study adenovirus human papillomavirus antigen immunogenicity (HPV) [28] modified to include two rounds of moDC co-culture, 48 h apart (Fig. 4A; Supplementary Fig. S8). Following two weeks of expansion using this method, we stimulated the cells with HERV-K-WT peptide pools, followed by sorting by IFN-γ capture and further expansion with IL-2 and irradiated feeder cells. 7 days post sorting, we saw evidence of a polyclonal CD8^+^ T cell response (Fig. 4B, left panel) most prominently in the groups expanded using moDCs transduced with HERV-K-ISDmut vectors. The CD4^+^ T cell responses showed a similar trend, but a single strong CD4^+^ T cell response in one of the HERV-K-WT-stimulated donor cultures made the distinction less apparent (Fig. 4B, right panel). 14 days after sorting, one of the strongest responders observed on day 7 had lost responsiveness, despite this, cultures stimulated with the HERV-K-ISDmut constructs still showed the strongest overall CD8^+^ and CD4^+^ T cell responses (Supplementary Fig. S9).

To explore if the HERV-K transduced moDC expanded T cells could recognize human cancer cells expressing HERV-K, we HLA typed our internally available cancer cell lines, and the HERV-K expanded donors, localizing a match between our donor 12 and the HCC827 cell line on the HLA-A11:01:01G allele (Fig. 4C). After incubating the HCC827 cells with HERV-K-ISDmut expanded T cells from donor 12, we observed antigen recognition measured by increased IFN-γ and TNF-α production in CD8^+^ T cells at a similar level to the peptide induced response, which was partially reversed by blocking with anti-HLA class I antibodies (Fig. 4D).

### In vivo immunogenicity of HERV-K-ISDmut delivered as adenovirus and mRNA

**Fig. 5:**
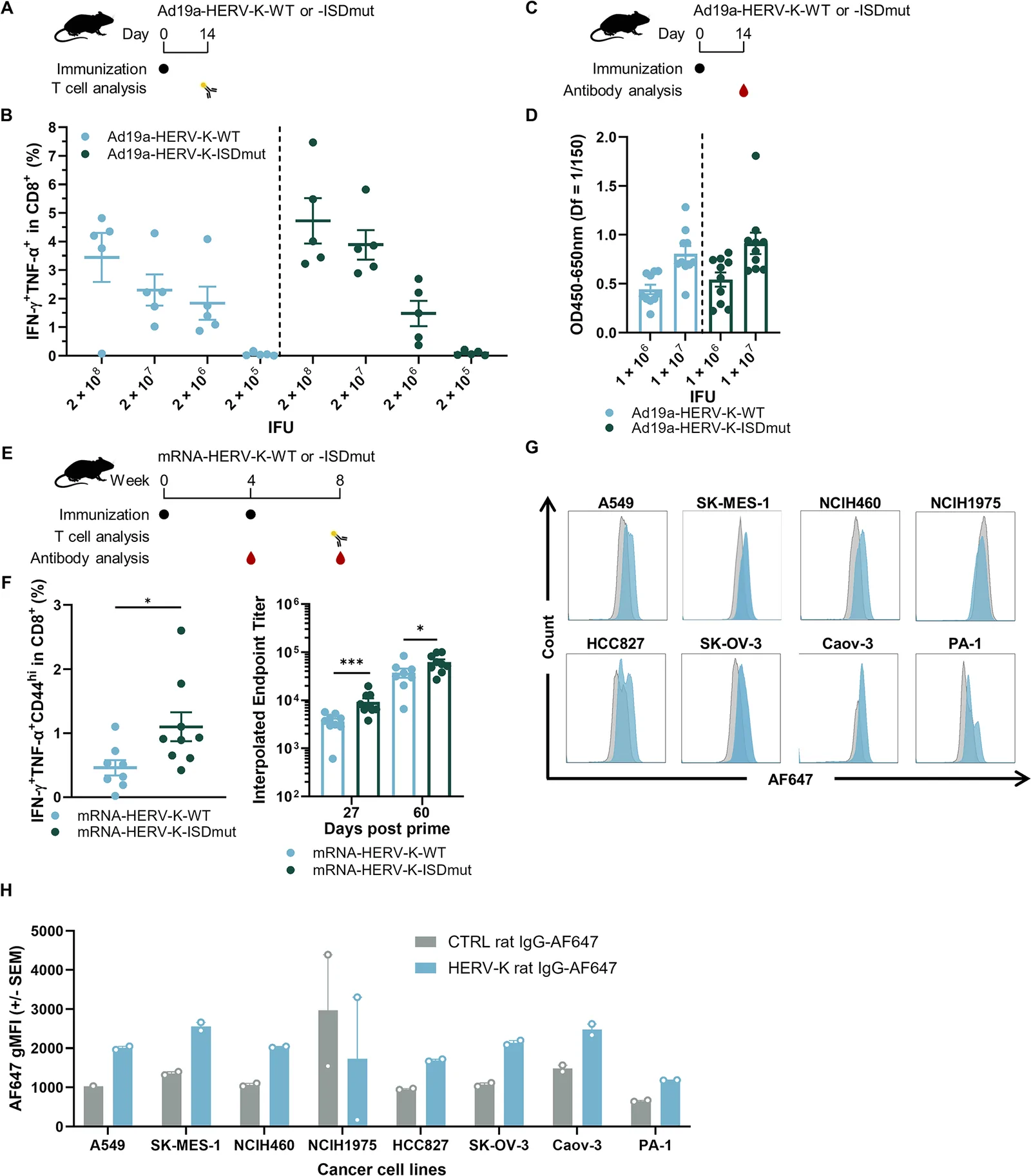
Adenoviral and mRNA–encoded HERV-K antigens elicit humoral and T cell immunity in mice. **A**, Protocol outline (created with BioRender.com). BALB/c mice (*n* = 5) immunized s.c. (footpad) on day 0 with de-escalating doses of Ad19a-HERV-K-WT or Ad19a-HERV-K-ISDmut. **B**, Flow-cytometric analysis of splenocytes collected as outlined in **A**, stimulated with ENV specific peptide (peptide 18), and stained intracellularly for T cell responses measured as the frequency of IFN-γ^+^, TNF-α^+^ double-positive cells. **C**, Protocol outline (created with BioRender.com). A separate cohort of BALB/c mice (*n* = 10) were immunized s.c. (hock) on day 0 with Ad19a-HERV-K-WT or Ad19a-HERV-K-ISDmut. **D**, ELISA analysis of serum collected as outlined in **C**. The graph shows analysis of antibody binding to the HERV-K ENV surface unit (SU) (dilution 1:150). **E**, Protocol outline (created with BioRender.com). BALB/c mice (*n* = 9) were immunized with 1.5 µg mRNA encoding either HERV-K-WT or HERV-K-ISDmut i.m. (right and left hind legs). One mouse from the WT group disappeared between prime and boost. **F**, (Left panel) flow-cytometric analysis of splenocytes collected as outlined in **E** stimulated with an ENV specific peptide (peptide 18) and stained intracellularly for T cell response measured as the frequency of IFN-γ^+^, TNF-α^+^ double-positive cells. (Right panel) ELISA analysis of serum collected as outlined in **E**. The graph shows analysis of antibody binding to HERV-K ENV surface unit (SU). **G**, Representative histograms for each cell line and condition shown in **H**. Blue and grey represent staining with IgG isolated from serum from rats immunized with either a vector encoding HERV-K or a control vector, respectively. **H**, Flow-cytometric analysis of HERV-K expression in different human cancer cell lines using intracellular staining with IgG isolated from serum from rats immunized with either a vector encoding HERV-K or a control vector (see Supplementary Fig. S11) labelled with AF647. Bars represent gMFI values for each condition. *P < 0.05 and ***P < 0.001 by Mann Whitney *U* test

To investigate HERV-K-WT and HERV-K-ISDmut immunogenicity differences in vivo, we immunized BALB/c mice with de-escalating dosages of adenoviral-vectored HERV-K-WT and HERV-K-ISDmut. A single dose induced strong CD8^+^ T cell responses at dosages as low as 2 × 10⁶ infectious units (IFU), but with no clear differences between Ad19a-HERV-K-WT and Ad19a-HERV-K-ISDmut in either cellular responses (Fig. 5A and B) or humoral responses (Fig. 5C and D) contrasting with the protein immunization results. As adenoviruses induce potent expression from a single transduction event, we investigated whether there was a potential immunogenicity threshold effect by also immunizing with the HERV-K-WT and HERV-K-ISDmut antigens as mRNAs formulated in lipid nanoparticles (LNPs). This re-introduced the significant difference in immunogenicity between HERV-K-WT and HERV-K-ISDmut constructs in both T cell and B cell responses (Fig. 5E and F), with the total magnitude of measured immune responses being lower when using mRNA vectors compared with adenoviral vectors. Given the differences between HERV-K-WT and HERV-K-ISDmut expressing human moDCs, the absence of difference in immunogenicity with adenoviruses in mice was a surprise, but we were likewise incapable of detecting differences in antigen expression and survival in HERV-K-WT and HERV-K-ISDmut expressing mouse Bone-Marrow-derived DCs (not shown).

To test the cancer recognition properties of HERV-K-ISDmut induced antibodies, we evaluated whether the antigen-specific antibodies could bind HERV-K-expressing cancer cells. This was assessed by flow cytometry and Western blot using serum from rats immunized with Ad19a-HERV-K-ISDmut and Ad5F35-HERV-K-ISDmut, or serum from Ad19a-CTRL and Ad5F35-CTRL immunized rats (Supplementary Fig. S11). Antibodies specifically bound HERV-K expressed on cancer cell lines measured by flow cytometry (Fig. 5G and **H**) and Western blot (Supplementary Fig. S12).

### Immunogenicity of HERV-K-ISDmut in transgenic mice and non-human primates

**Fig. 6:**
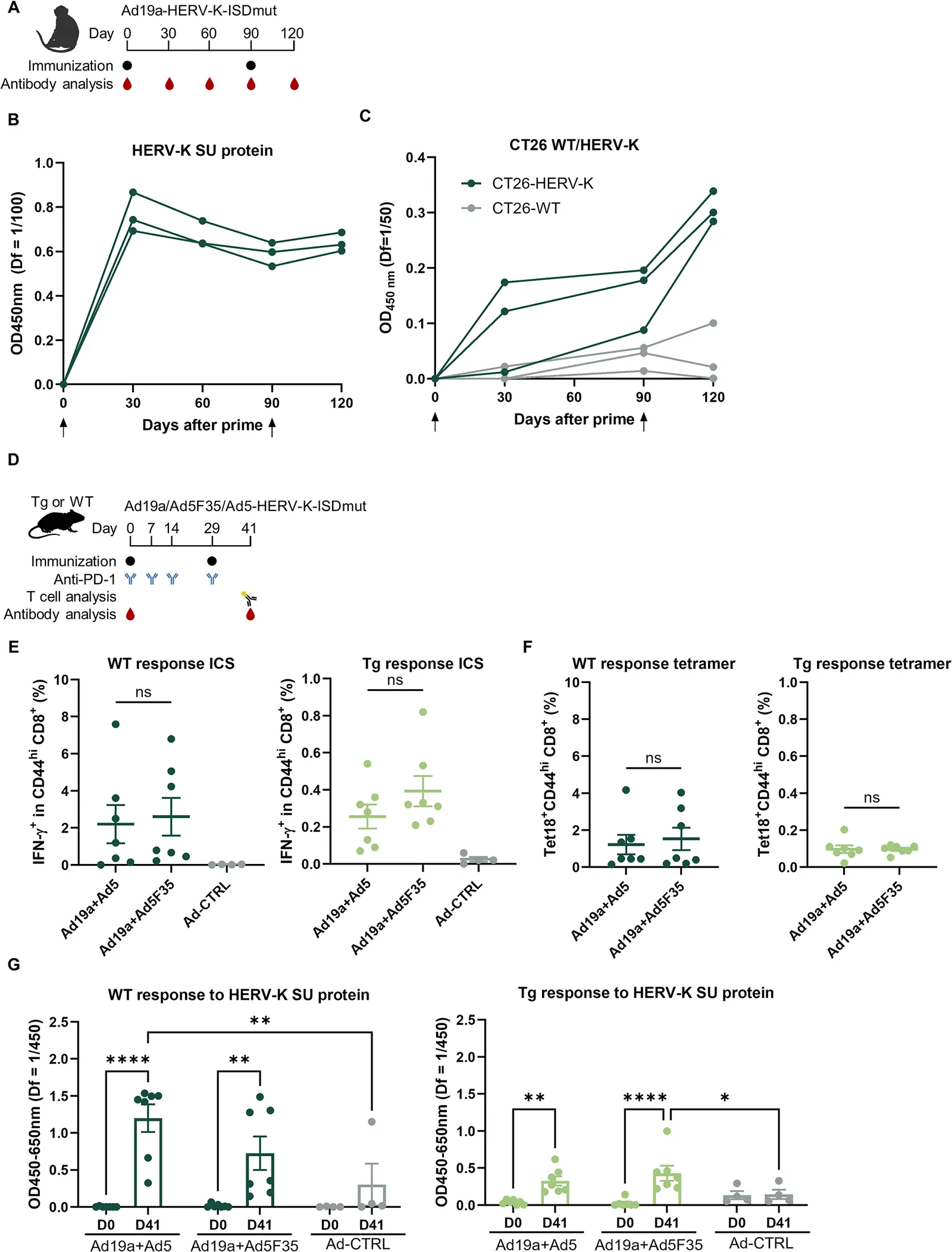
Adenoviral HERV-K-ISDmut immunization induces responses in cynomolgus macaques and HERV-K transgenic mice. **A**, Protocol outline (created using BioRender.com). Cynomolgus macaques (*n* = 3) were immunized with 2 × 10^9^ IFU Ad19a-HERV-K-ISDmut i.m. on day 0 and 90. Blood samples collected on days 0, 30, 60, 90, and 120. **B**, ELISA analysis of serum from cynomolgus macaques, collected as outlined in **A**, for antibody binding of HERV-K ENV surface unit (SU, dilution 1:100). **C**, Cell-binding assay analysis of serum from cynomolgus macaques collected as outlined in **A** for binding to CT26-HK (dilution 1:50). **D**, Protocol outline (created using BioRender.com). CB6F1 WT (WT) littermates and CB6F1 HERV-K Transgenic (Tg) mice (generated as in Supplementary Fig. S13) were primed on day 0 with 2 × 10⁸ IFU Ad19a-HERV-K-ISDmut s.c. (hock) and boosted on day 29 with either 2 × 10⁷ IFU Ad5-HERV-K-ISDmut s.c. (hock) or 2 × 10⁷ IFU Ad5F35-HERV-K-ISDmut s.c. (hock). Anti-PD-1 (200 µg, i.p.) was administered on days 0, 7, 14, and 29. Tg and WT control mice were immunized with 2 × 10⁸ IFU empty Ad19a and Ad5 s.c. (hock) on day − 28 and day 29, respectively. Anti-PD-1 (200 µg, i.p.) was administered on days − 28, -20, -13, and 0. Spleens were harvested on day 41. **E**, Flow-cytometric analysis of splenocytes collected as outlined in **D**, stimulated with ENV specific peptide 18, and stained intracellularly for T cell response measured as the frequency of IFN-γ^+^ cells. **F**, Flow-cytometric analysis of splenocytes collected as outlined in **D**, stained with a tetramer bound by HERV-K ENV-specific peptide 18. **G**, ELISA analysis of serum collected as outlined in **D** for antibody binding to the HERV-K ENV surface unit (SU, dilution 1:450). *P < 0.05, **P < 0.01, and ****P < 0.0001 by two-way ANOVA with Tukey’s multiple-comparison test

To test if Ad19a-HERV-K-ISDmut could elicit immune responses in a highly translationally relevant model, we immunized cynomolgus macaques with Ad19a-HERV-K-ISDmut, followed by a boost with the same vector on day 90 (Fig. 6A). The priming immunization with Ad19a-HERV-K-ISDmut rapidly raised HERV-K-specific antibodies, capable of binding the surface unit (SU) of the HERV-K ENV protein (Fig. 6B). The homologous boost modestly increased antibody titers against the protein and further increased responses in the cell-binding assay using CT26-HK cells (Fig. 6C). However, although macaques have shared ERV-K integrations, many ERV-K sequences in cynomolgus macaques and humans are no longer identical [40, 41]. To further evaluate the in vivo immunogenicity of Adenovirus-encoded HERV-K-ISDmut in a setting of partial tolerance, we generated a transgenic mouse line expressing the WT HERV-K *gag* and *env* sequences under the control of the constitutive mouse mammary tumor virus (MMTV) promoter (Supplementary Fig. S13A and B for transgene design, Supplementary Fig. S13C for characterization). As anti-PD1 treatment had augmented responses in transgenic and WT mice (Supplementary Fig. S14A), we tested Ad19a with either Ad5 or Ad5F35 booster with anti-PD1 in HERV-K transgenic and HERV-K littermate controls (Fig. 6D-G) confirming the induction of T cell immunity against HERV-K ENV peptide 18 and a serological response, both of which were higher in WT mice than in transgenic mice. No significant differences were observed between Ad5 and Ad5F35 boosts at 12 days post-boost (day 41) in cellular responses to HERV-K ENV peptide 18 (Fig. 6E) or in tetramer staining (Fig. 6F). Similarly, differences in B cell responses were not significant (Fig. 6G). Assessment of T cell memory at 40 days post-boost (day 69), confirmed persistent T and B cell responses, with a stronger antibody response after Ad5F35 immunization in WT mice (Supplementary Fig. S14B).

### HERV-K-ISDmut immunization improves survival and limits experimental metastases of HERV-K expressing Renca cells in mice

**Fig. 7:**
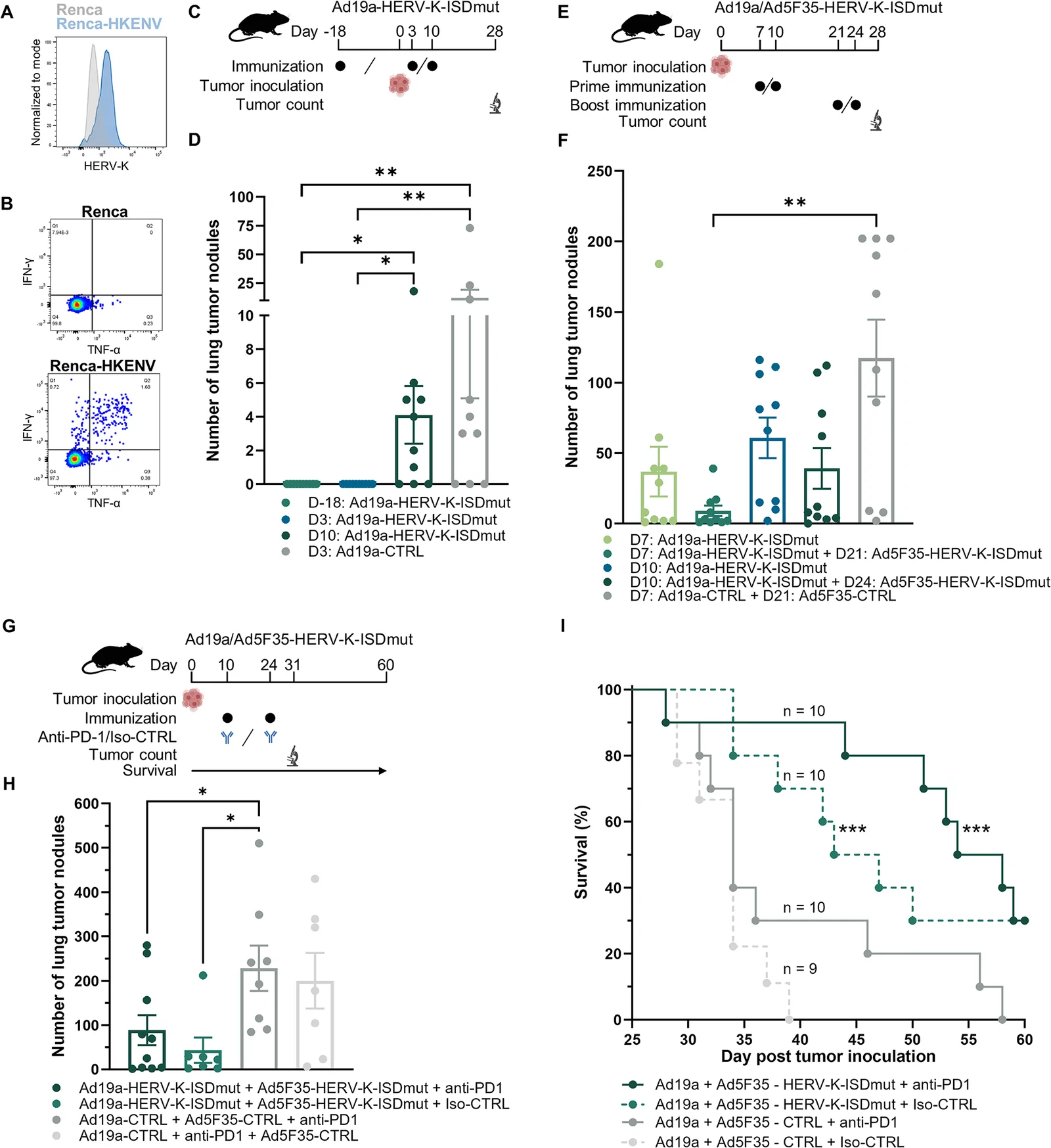
Adenoviral HERV-K-ISDmut immunization induces responses that provide tumor control. **A**, Flow-cytometric analysis of Renca-HKENV (blue) and WT Renca cells (grey) stained with ENV specific antibody HERM-1811-5 labelled with AF647. **B**, Spleens from mice (*n* = 3) immunized with 2 × 10⁸ IFU Ad19a-HERV-K-ISDmut s.c. (hock) on day 0, and 2 × 10⁸ IFU Ad5-HERV-K-ISDmut s.c. (hock) on day 28 were harvested 11 days post-boost. Graphs show flow-cytometric analysis of IFN-γ^+^ and TNF-α^+^ in CD8^+^ splenocytes stimulated with Renca or Renca-HKENV cells. **C**, Protocol outline (created using BioRender.com). BALB/c mice (*n* = 10) were immunized with 2 × 10⁸ IFU Ad19a-HERV-K-ISDmut s.c. (hock) on day − 18, 3, or 10; or with Ad19a-CTRL (empty vector) s.c. (hock) on day 3. Mice were inoculated with Renca-HKENV cells (i.v.) on day 0. Lungs were harvested on day 28. **D**, Tumor nodule counts in lungs harvested as outlined in **C**. **E**, Protocol outline (created using BioRender.com). BALB/c mice (*n* = 10 per group) were inoculated with Renca-HKENV cells (i.v.) on day 0. Mice were primed with 2 × 10⁸ IFU Ad19a-HERV-K-ISDmut s.c. (hock) on day 7 or 10 and boosted with 2 × 10⁷ IFU Ad5F35-HERV-K-ISDmut s.c. (hock) on day 21 or 24. Control mice were primed s.c. (hock) with 2 × 10⁸ IFU Ad19a-CTRL (empty vector) on day 7 and boosted with 2 × 10⁷ IFU Ad5F35-CTRL (irrelevant antigen) s.c. (hock) on day 21. Lungs were harvested on day 28. **F**, Tumor nodule counts in lungs harvested as outlined in **E**. **G**, Protocol outline (created using BioRender.com). BALB/c mice (*n* = 7–10 per group) were inoculated with Renca-HKENV cells (i.v.) on day 0. Mice were primed with 2 × 10⁸ IFU Ad19a-HERV-K-ISDmut s.c. (hock) on day 10 and boosted with 2 × 10⁷ IFU Ad5F35-HERV-K-ISDmut s.c. (hock) on day 24. Control mice were primed s.c. (hock) with 2 × 10⁸ IFU Ad19a-CTRL (irrelevant antigen) on day 10 and boosted with 2 × 10⁷ IFU Ad5F35-CTRL (irrelevant antigen) s.c. (hock) on day 24. Mice were administered either anti-PD-1 (200 µg, i.p.) or isotype control antibody (200 µg, i.p.) on day 24, except one group that was administered anti-PD-1 on day 10 (light grey, **H**). **H**, Tumor nodule counts in lungs harvested as outlined in **G**. **I**, Graph shows overall survival of mice treated as outlined in **G**. *P < 0.05, **P < 0.01, and ***P < 0.001. Kruskal-Wallis test followed by Dunn’s multiple-comparison test and survival curves are log-rank (Mantel-Cox) tests

To test the adenoviral vector HERV-K-ISDmut prime/boost regimen in an in vivo tumor model, we used a BALB/c derived renal carcinoma cell line expressing HERV-K ENV as a foreign antigen (Renca-HKENV) [42]. HERV-K ENV expression on Renca-HKENV was confirmed by cell surface staining using the HERV-K specific monoclonal antibody HERM-1811-5 (Fig. 7A) and CD8^+^ T cells from HERV-K-ISDmut immunized BALB/c mice, showed increased responses when stimulated with Renca-HKENV cells compared to WT Renca cells (Fig. 7B).

Prophylactic and therapeutic immunization with Ad19a-HERV-K-ISDmut alone, either 18 days before, or 3 or 10 days after tumor challenge (Fig. 7C), prevented and eliminated early growing tumors, but a reduction of tumor burden with immunization 10 days post challenge did not achieve statistical significance (Fig. 7D). A repeat with therapeutic immunizations on day 7 or day 10, with or without an accelerated boost (Ad5F35-HERV-K-ISDmut) (Fig. 7E) reduced tumor burden in all immunized groups although only the HERV-K-ISDmut prime-boost immunization on day 7 induced a statistically significant reduction in tumor burden due to extensive variability (Fig. 7F). Accordingly, we selected the toughest prime-boost regimen initiated on day 10 after tumor challenge to evaluate tumor control and survival in combination with anti-PD-1 therapy (Fig. 7G). HERV-K-ISDmut prime-boost therapeutic immunization significantly improved clearance of metastases compared to late anti-PD-1 monotherapy in control immunized mice (Fig. 7H). In the survival analysis, HERV-K-ISDmut prime-boost significantly improved survival compared to the control group with or without anti-PD-1 whereas anti-PD-1 monotherapy in control immunized mice did not (Fig. 7I).

## Discussion

Mouse γ-retrovirus ERVs have long been recognized as highly effective anti-cancer targets, and we have previously been able to exploit ISD mutated mouse ERVs as targets in syngeneic mouse cancer models [4]. There is, however, a translational challenge in extrapolating findings from mouse γ-retrovirus ERVs to human cancer-associated HERV-K β-retrovirus ERVs, as they differ substantially in their structural integrity. Also, despite the expression of other HERVs in cancers, HERV-K is the only ERV confirmed to produce retrovirus-like particles [43]. The relative structural integrity of HERV-K, together with the expression of secreted ENV proteins, creates the potential to elicit coordinated B cell, CD4^+^ T cell and CD8^+^ T cell responses directed against the same HERV-K antigen expressed by tumor cells, which might not be possible for many other cancer-expressed antigens. This potentially explains the association between HERV-K immunogenicity and responses to immunotherapy in patients and highlights the opportunity in enhancing anti-HERV-K immunity in patients [44]. Here, we further add to this potential by demonstrating that HERV-K is widely expressed at the RNA level in most patients in most cancer types in a large cohort of patients admitted to a phase 1 trial unit. Although our cohort lacks healthy control samples, the data can be interpreted together with an extensive array of studies finding HERV-K expression elevated in cancers [7, 9, 10, 15–17, 29, 30], including a cohort study using relatively similar sequencing methods in breast cancer patients which is not an unusually high expression cancer type in our dataset [30]. Interpreted in light of the previous studies, our findings confer generality to the reports of HERV-K cancer association [7, 8] and extend these observations to pre-treated patients that are the most relevant for early immunotherapeutic development.

To elicit potent HERV-K-specific B cell and T cell immunity we used adenoviral vectors co-expressing HERV-K/HML-2 GAG and ENV consensus antigens similarly to the previous mouse ERV vaccines. This particular antigen design enabled VLP formation capable of inducing CD4^+^ T cell and B cell responses, while also enabling direct antigen presentation through MHC-I pathways [45]. We previously showed that mutation of the ISD was required for robust immunogenicity [4]. Here, we screened a previously hypothesized [31], but independently inactive ISD domain [18] for mutations that increased immunogenicity rather than assessing immune suppressive function. We evaluated single amino acid substitutions within the ISD for pro-inflammatory signaling using an NF-κΒ reporter assay and identified the Q525A substitution as the most potent activator. To our knowledge, the Q525A mutation is the first immunostimulatory ISD mutation described for β-retroviruses and is not co-linear with the previously described ISD mutation in γ-retroviruses [31]. In contrast, it matches an HIV ISD mutation previously characterized by Morozov et al., which was found to enhance B cell immunogenicity in mouse models compared with the WT sequence [46]. The HIV ISD mutation by Morozov et al., preserved fusion activity, and it still increased immune responses towards the WT antigen in animals immunized with an HIV gp41 extracellular domain, just as HERV-K-ISDmut enhanced immunogenicity in our TM protein immunization assay. At the transcriptional level, our datasets indicate that the HERV-K ENV ISD mutation shifts the transcriptional program induced by the WT HERV-K sequence from cellular senescence towards antigen presentation and programmed cell death associated pathways, a finding that despite robust activation of death pathways was not impeded by sample quality and/or progression of cell death as evidenced by similar yield, RNA RIN score and principal component analysis which highlight vector identity as the principal variable in the donor adjusted PCA analysis. With the abrogated fusion activity of the expression cassette this would be an important safety parameter for its application as an immunotherapy agent in patients. Reduced senescence associated pathways by the ISD mutation are further consistent both with the non-redundant role of the HERV-K ENV protein in cellular senescence [38] and with the increased human T cell expansion we observed using the ISDmut antigen in moDC-PBMC co-cultures in matched expansions with PBMCs from 4 individual donors. It also provides an explanation for a net immune suppressive function of the native ISD domain in context of broader functional properties of the ENV protein, as opposed to simply a disabled peptide domain with independent immune suppressive functions. Importantly, our results agree with published data in that the human T cell repertoire includes T cells of sufficiently high avidity to recognize HERV-K expressing human cancer cell lines [10, 47]. Concerning the expansion of human T cells, we show here that the HERV-K-ISDmut vectors can expand not only CD8^+^ T cells, but also in most cases CD4^+^ T cells, and we show that the PBMCs and the induced CD8^+^ T cells can directly recognize a human HLA matched NSCLC cell line. Unfortunately, the T cell expansion assays are cumbersome and the re-expansions from frozen cultures are not always successful. This is the reason for the limited number of cultures and cancer cell recognition data, which further limited the number of positive T cell and cancer matches to a single successful example with some HLA-A2*0101 matches not resulting in T cell stimulation, suggesting that T cell responses were restricted to other HLA molecules. The ability to expand CD4^+^ T cells in moDC and PBMC cocultures is a welcomed surprise for an intracellularly expressed antigen and agrees with the RNAseq gene set enrichment signatures that show significant increased autophagy and MHC class II antigen presentation pathways, although secretion and re-uptake of antigen during the cultures cannot be ruled out as a mechanism. Studies in mouse models confirmed these findings, demonstrating potent B and T cell responses as well as cancer protection in WT mice, and break of tolerance, albeit at lower response levels in transgenic mice. To supplement these experiments, we also included immunizations of 3 cynomolgus macaques which demonstrated B cell responses as compared to pre-immunizations controls. However, it should be acknowledged that these studies lacked a group to control for unspecific immune activation by the vector or a HERV-K-WT group. These limitations make it impossible to prove the ISD mutation was involved in immunogenicity, and the sequence diversity between human and macaque ERV-K makes it impossible to claim that tolerance had been broken against the macaque ERV-K. The use of a pseudotyped cell line is a necessity for validating a human antigen expressing immunotherapy in immune competent models and further allowed us to encapsulate the improvement of efficacy by anti-PD1 monoclonal antibody treatment. Nevertheless, it does not address if similar immunity can be induced in humans, nor does a transplantable model recapitulate a natural human tumor type. The study lacks a demonstration of T and B cell synergy and interaction, which in human cancers would additionally involve the generation of tertiary lymphoid structures. However, this is a finding of human anti-tumor immunity not recapitulated in transplantable animal syngeneic tumor models expressing foreign antigens [48]. Other classical models used in validating immunotherapeutic approaches such as PDX models, are still fundamentally incapable of studying induced tumor antigen specific T and B cell immunity. For future potential in human cancer treatment, the most important implication of the ISD induced increase in CD4^+^ and CD8^+^ T cell immunogenicity, is not the ability to induce relevant levels of T cell immunity per se, but the ability to do so with what is a structurally intact tumor associated B cell antigen presenting the native pre-fusion non-linear epitopes. In recent years it has become increasingly clear that tumor protection relies on T cell killing, which in turn depends on antigen presentation networks including helper T cells and B cells often seen within the tumor parenchyma [49–51]. As HERV-K is a naturally immunogenic B cell antigen linked to immune checkpoint inhibitor responses [44], with incorporation into tumor secreted extracellular vesicles [52], and the display of ENV proteins on cancer cell surfaces [29], the described anti-HERV-K therapy may have special potential in supporting such cellular interactions in cancer patients. The potential ability to support immune interaction networks of non-redundant nature towards a self-antigen, naturally raises the question of whether such immunity can be safely induced in humans. As computationally confirmed (Supplementary Fig. S2), our antigen may induce responses towards a wide range of HERV-K provirus encoded proteins, structurally or sequentially related epitopes on different ERV families, as well as random cross-reactivity against conventional host proteins. This will always be a safety concern for any new antigen and should be addressed in clinical trials. Nevertheless, HERV-K’s prominent involvement in cancers has resulted in numerous studies detecting T and B cell immune responses towards HERV-K without associated toxicity [7, 10, 15, 17, 29, 47]. Similarly, previous analysis of antibody responses to self-antigens [53] and characterization of T cell epitopes [10] further support the safety of HERV-K-specific immunity.

Despite the improved B and T cell immunogenicity of the ISD mutated HERV-K protein and mRNA vector compared to WT ISD, we did not observe a comparable enhancement in immunogenicity when the mutated ISD antigen was delivered using adenoviral vectors. Species-specific effects of ISD function and ISD mutations have previously been described for γ-retroviruses and have been proposed to arise from interactions with cognate host-cell receptors [54]. We found that co-expression of HERV-K ENV with HERV-K GAG diminished cell fusion capability, but this does not mitigate the possibility that cellular receptor interactions play a role in the HERV-K-WT and HERV-K-ISDmut immunogenicity. This may not be adequately recapitulated in the mouse model, which could partially explain the reduced effect of the ISD mutation in the mouse model if it has less preserved interaction partners. A reduced effect in mouse cells might not be important with adenoviruses that exhibit strong antigen expression and could potentially saturate a reduced receptor interaction space in non-host murine cells, diminishing the interactions that may be responsible for the robust effects in human cells. In contrast, lower and variable levels of antigen expression can be expected with mRNA vectors and after in vivo uptake of recombinant protein in adjuvant, which may account for lower, but enhanced immune responses observed with the ISD mutated antigens.

## Conclusions

Overall, the data presented here, including augmented human T cell expansion and B cell responses in non-human primates, the extensive body of literature demonstrating HERV-K immunogenicity and tumor targeting capability of the T cell repertoire, suggest that HERV-K antigens can be engineered for increased immunogenicity, enabling the translation of previously pre-clinically successful cancer immunotherapy technologies.

## Supplementary Information

Below is the link to the electronic supplementary material.

Supplementary Material 1

Supplementary Material 2

## Data Availability

All data associated with this study are present in the paper or the Supplementary Materials. The human data from the human study [19] are sensitive in nature as they include human subject data, and therefore, the data cannot be made publicly available. TEM images and Flow cytometry standard (FCS) files and associated gating strategies are available from Anne-Marie C. Andersson and Peter J. Holst upon reasonable request. Cell lines, plasmids, viral vectors, and engineered HERV-K constructs described in this study are available under a material transfer agreement (MTA) and may require additional institutional approvals. Access to non-human primate samples and human PBMC biospecimens is subject to ethical approval and material availability and may require additional agreements. Requests for materials should be directed at Anne-Marie C. Andersson and Peter J. Holst.

## Abbreviations

Ad19a: Adenovirus type 19a/64
Ad5: Adenovirus type 5
Ad5F35: Chimeric adenovirus type 5/F35
AML: Acute myeloid leukaemia
BCA: Bicinchoninic acid
CPM: Counts per million
CoPPO: Copenhagen Prospective Personalized Oncology
CUP: Cancer of unknown primary
DC: Dendritic cell
DEG: Differentially expressed gene
ELISA: Enzyme-linked immunosorbent assay
ENV: Envelope protein
ERV: Endogenous retrovirus
FACS: Fluorescence-activated cell sorting
GAG: Group-specific antigen
Gastric_GEJ: Gastric/Gastroesophageal junction cancer
GSEA: Gene set enrichment analysis
HERV: Human endogenous retrovirus
HERV-K: Human endogenous retrovirus K
HLA: Human leukocyte antigen
HML: Human MMTV-like
HRP: Horseradish peroxidase
ICS: Intracellular cytokine staining
IFN: Interferon
IFU: Infectious units
IL: Interleukin
ISD: Immunosuppressive domain
LNP: Lipid nanoparticle
MANEC: Mixed adeno-neuroendocrine carcinoma
MiNEN: Mixed neuroendocrine–non-neuroendocrine neoplasm
MHC: Major histocompatibility complex
MLV: Murine leukemia virus
MOI: Multiplicity of infection
NEC: Neuroendocrine carcinoma
NET: Neuroendocrine tumor
NF-κB: Nuclear factor kappa B
NSCLC: Non-small cell lung cancer
NUT carcinoma: Nuclear Protein in Testis (NUT) Midline Carcinoma
P2A: Porcine teschovirus-1 2 A peptide
PBMC: Peripheral blood mononuclear cell
PFA: Paraformaldehyde
RNA-seq: RNA sequencing
SCLC: Small cell lung cancer
STAR: Spliced Transcripts Alignment to a Reference
SU: Surface unit
TEM: Transmission electron microscopy
TM: Transmembrane
VLP: Virus-like particle
WT: Wild type
gMFI: Geometric mean fluorescence intensity
moDC: Monocyte-derived dendritic cell

## References

[1] Laumont CM, Banville AC, Gilardi M, Hollern DP, Nelson BH. Tumour-infiltrating B cells: immunological mechanisms, clinical impact and therapeutic opportunities. Nat Rev Cancer [Internet] Nat Res. 2022;22:414. 10.1038/S41568-022-00466-1. [cited 2026 Jun 2];. PMC9678336 35393541

[2] Stein RA, Depaola RV. Human endogenous retroviruses: our genomic fossils and companions. Am Physiol Soc Rockv, MD; 2023;55:249–58. 10.1152/physiolgenomics001712022. 37154499

[3] Kang BH, Momin N, Moynihan KD, Silva M, Li Y, Irvine DJ, et al. Immunotherapy-induced antibodies to endogenous retroviral envelope glycoprotein confer tumor protection in mice. PLoS One [Internet] PLoS One. 2021. 10.1371/JOURNAL.PONE.0248903. [cited 2026 Jun 3];16. PMC8049297 33857179

[4] Daradoumis J, Ragonnaud E, Skandorff I, Nielsen KN, Bermejo AV, Andersson AM, et al. An Endogenous Retrovirus Vaccine Encoding an Envelope with a Mutated Immunosuppressive Domain in Combination with Anti-PD1 Treatment Eradicates Established Tumours in Mice. Viruses [Internet] Viruses. 2023. 10.3390/V15040926. [cited 2026 May 11];15. PMC10141008 37112906

[5] Ueda MT, Kryukov K, Mitsuhashi S, Mitsuhashi H, Imanishi T, Nakagawa S. Comprehensive genomic analysis reveals dynamic evolution of endogenous retroviruses that code for retroviral-like protein domains. Mob DNA [Internet]. 2020;11:29. 10.1186/s13100-020-00224-w. PMC7499964 32963593

[6] Montserrat-Ayuso T, Esteve-Codina A. A Comprehensive annotation of conserved protein domains in human endogenous retroviruses [Internet]. 2025. 10.1101/2025.07.25.666750. PMC12884080 41669668

[7] Müller MD, Holst PJ, Nielsen KN. A Systematic Review of Expression and Immunogenicity of Human Endogenous Retroviral Proteins in Cancer and Discussion of Therapeutic Approaches. Int J Mol Sci [Internet]. 2022;23:1330. 10.3390/ijms23031330. PMC8836156 35163254

[8] Stricker E, Peckham-Gregory EC, Scheurer ME, CancerHERVdb. Human endogenous retrovirus (HERV) Expression database for human cancer accelerates studies of the retrovirome and predictions for HERV-based therapies. Simon V, editor. J Virol [Internet]. 2023;97:e0005923. 10.1128/jvi.00059-23. PMC10308937 37255431

[9] Jiang Q, Braun DA, Clauser KR, Ramesh V, Shirole NH, Duke-Cohan JE, et al. HIF regulates multiple translated endogenous retroviruses: Implications for cancer immunotherapy. Cell [Internet]. 2025;188:1807–e182734. 10.1016/j.cell.2025.01.046. PMC11988688 40023154

[10] Bonaventura P, Page A, Tabone O, Estornes Y, Mutez V, Delles M, et al. HERV-derived epitopes represent new targets for T-cell-based immunotherapies in ovarian cancer. J Immunother Cancer [Internet]. 2025;13:e010099. 10.1136/jitc-2024-010099. PMC12320039 40759443

[11] Kobayashi S, Tokita S, Moniwa K, Kitahara K, Iuchi H, Matsuo K, et al. Proteogenomic identification of an immunogenic antigen derived from human endogenous retrovirus in renal cell carcinoma. JCI Insight [Internet]. 2023;8. 10.1172/jci.insight.167712. PMC10543709 37606040

[12] Neukirch L, Nielsen TK, Laursen H, Daradoumis J, Thirion C, Holst PJ. Adenovirus based virus-like-vaccines targeting endogenous retroviruses can eliminate growing colorectal cancers in mice. Oncotarget [Internet]. 2019;10:1458–72. 10.18632/oncotarget.26680. PMC6402721 30858929

[13] Skandorff I, Ragonnaud E, Gille J, Andersson AM, Schrödel S, Duvnjak L, et al. Human Ad19a/64 HERV-W Vaccines Uncover Immunosuppression Domain-Dependent T-cell response differences in inbred mice. Int J Mol Sci Multidisciplinary Digit Publishing Inst (MDPI). 2023;24. 10.3390/ijms24129972. PMC10297909 37373123

[14] Belshaw R, Dawson ALA, Woolven-Allen J, Redding J, Burt A, Tristem M. Genomewide screening reveals high levels of insertional polymorphism in the human endogenous retrovirus family HERV-K(HML2): implications for present-day activity. J Virol [Internet]. 2005;79:12507–14. 10.1128/JVI.79.19.12507-12514.2005. PMC1211540 16160178

[15] Wang-Johanning F, Radvanyi L, Rycaj K, Plummer JB, Yan P, Sastry KJ, et al. Human endogenous retrovirus K triggers an antigen-specific immune response in breast cancer patients. Cancer Res [Internet]. 2008;68:5869–77. 10.1158/0008-5472.CAN-07-6838. PMC5802396 18632641

[16] Attig J, Pape J, Doglio L, Kazachenka A, Ottina E, Young GR, et al. Human endogenous retrovirus onco-exaptation counters cancer cell senescence through calbindin. J Clin Invest [Internet]. 2023;133. 10.1172/JCI164397. PMC10348765 37192000

[17] Nguyen QTT, Shin SG, Nguyen TTT, Lee KY, McClelland M, Lee EJ. Human endogenous retrovirus-K envelope protein is aberrantly expressed in serous ovarian cancer and promotes chemosensitivity via NF-κB/P-glycoprotein pathway inhibition. J Ovarian Res [Internet]. BioMed Central Ltd; 2025 [cited 2026 Jun 12];18:146. 10.1186/S13048-025-01722-2. PMC12232173 40616136

[18] Bahrami S, Gryz EA, Graversen JH, Troldborg A, Stengaard Pedersen K, Laska MJ. Immunomodulating peptides derived from different human endogenous retroviruses (HERVs) show dissimilar impact on pathogenesis of a multiple sclerosis animal disease model. Clin Immunol [Internet]. 2018;191:37–43. 10.1016/j.clim.2018.03.007. 29567431

[19] Belcaid L, Højgaard M, Tuxen I, Spanggaard I, Lassen U, Robinson L, et al. Copenhagen Prospective Personalized Oncology (CoPPO)-impact of comprehensive genomic profiling in more than 2000 patients in a phase I setting. Ann Oncol [Internet] Elsevier. 2025;36:1078–87. 10.1016/j.annonc.2025.04.004. 40246201

[20] Subramanian RP, Wildschutte JH, Russo C, Coffin JM. Identification, characterization, and comparative genomic distribution of the HERV-K (HML-2) group of human endogenous retroviruses. Retrovirology [Internet]. 2011;8:90. 10.1186/1742-4690-8-90. PMC3228705 22067224

[21] Montserrat-Ayuso T, Pujol A, Esteve-Codina A. Regulatory features and functional specialization of human endogenous retroviral LTRs: A genome-wide annotation and analysis via HERVarium. bioRxiv [Internet]. 2026;2026.02.17.706328. 10.64898/2026.02.17.706328.

[22] Ruzsics Z, Lemnitzer F, Thirion C. Engineering adenovirus genome by bacterial artificial chromosome (BAC) technology. Methods Mol Biol [Internet]. 2014;1089:143–58. 10.1007/978-1-62703-679-5_11. 24132484

[23] Berg C, Daugvilaite V, Steen A, Jørgensen AS, Våbenø J, Rosenkilde MM. Inhibition of HIV fusion by small molecule agonists through efficacy-engineering of CXCR4. ACS Chem Biol [Internet]. 2018 [cited 2026 Aug 23];13:881–6. 10.1021/ACSCHEMBIO.8B00061. 29461034

[24] Baker JM, Boyce FM. High-throughput Functional Screening using a Homemade Dual-glow Luciferase Assay. J Vis Exp [Internet] J Visualized Experiments. 2014. 10.3791/50282. [cited 2026 Aug 23];50282. PMC4186351 24962249

[25] Kamala T. Hock immunization: a humane alternative to mouse footpad injections. J Immunol Methods [Internet]. 2007;328:204–14. 10.1016/j.jim.2007.08.004. PMC2464360 17804011

[26] Lee YN, Bieniasz PD. Reconstitution of an infectious human endogenous retrovirus. Holmes EC, editor. PLoS Pathog [Internet]. 2007;3:e10. 10.1371/journal.ppat.0030010 PMC1781480 17257061

[27] Janik P, Briand P, Hartmann NR. The effect of estrone-progesterone treatment on cell proliferation kinetics of hormone-dependent GR mouse mammary tumors. Cancer Res [Internet]. 1975;35:3698–704. http://www.ncbi.nlm.nih.gov/pubmed/1192428. 1192428

[28] Daradoumis J, Müller MD, Neckermann P, Asbach B, Schrödel S, Thirion C, et al. Preferential Expansion of HPV16 E1-Specific T Cells from Healthy Donors’ PBMCs after Ex Vivo Immunization with an E1E2E6E7 Fusion Antigen. Cancers (Basel) [Internet]. 2023;15:5863. 10.3390/cancers15245863. PMC10741473 38136407

[29] Wang-Johanning F, Rycaj K, Plummer JB, Li M, Yin B, Frerich K, et al. Immunotherapeutic potential of anti-human endogenous retrovirus-K envelope protein antibodies in targeting breast tumors. J Natl Cancer Inst [Internet] J Natl Cancer Inst. 2012;104:189–210. 10.1093/JNCI/DJR540. [cited 2026 May 10];. PMC3274512 22247020

[30] Wei Y, Wei H, Wei Y, Tan A, Chen X, Liao X, et al. Screening and identification of human endogenous retrovirus-k mrnas for breast cancer through integrative analysis of multiple datasets. Front Oncol [Internet]. Frontiers Media S.A. 2022 [cited 2026 Aug 23];12:820883. 10.3389/FONC.2022.820883/FULL. PMC8900282 35265522

[31] Morozov VA, Dao Thi VL, Denner J. The transmembrane protein of the human endogenous retrovirus–K (HERV-K) modulates cytokine release and gene expression. Belshaw R, editor. PLoS One [Internet]. 2013;8:e70399. 10.1371/journal.pone.0070399. PMC3737193 23950929

[32] Shaked R, Katz M, Cohen-Dvashi H, Diskin R. The prefusion structure of the HERV-K (HML-2) Env spike complex. Proceedings of the National Academy of Sciences [Internet]. 2025;122:e2505505122. 10.1073/pnas.2505505122. PMC12280955 40632556

[33] Shek J, Sun C, Wilson EM, Moadab F, Hastie KM, Rajamanickam RR, et al. Human endogenous retrovirus K (HERV-K) envelope structures in pre- and postfusion by cryo-EM. Sci Adv [Internet]. 2025;11. 10.1126/sciadv.ady8168. PMC12383274 40864726

[34] Nelson M, Nelson DS, Cianciolo GJ, Snyderman R. Effects of CKS-17, a synthetic retroviral envelope peptide, on cell-mediated immunity in vivo: immunosuppression, immunogenicity, and relation to immunosuppressive tumor products. Cancer Immunol Immunother [Internet]. 1989;30:113–8. 10.1007/BF01665962. PMC11038887 2598177

[35] Meng EC, Goddard TD, Pettersen EF, Couch GS, Pearson ZJ, Morris JH, et al. <scp>UCSF ChimeraX</scp>: Tools for structure building and analysis. Protein Sci [Internet]. 2023;32:e4792. 10.1002/pro.4792. PMC10588335 37774136

[36] Ragonnaud E, Schroedel S, Mariya S, Iskandriati D, Pamungkas J, Fougeroux C, et al. Replication deficient human adenovirus vector serotype 19a/64: Immunogenicity in mice and female cynomolgus macaques. Vaccine [Internet]. 2018;36:6212–22. 10.1016/j.vaccine.2018.07.075. 30190120

[37] Flickinger JC, Singh J, Carlson R, Leong E, Baybutt TR, Barton J, et al. Chimeric Ad5.F35 vector evades anti-adenovirus serotype 5 neutralization opposing GUCY2C-targeted antitumor immunity. J Immunother Cancer [Internet]. 2020;8:e001046. 10.1136/jitc-2020-001046. PMC7443303 32819976

[38] Liu X, Liu Z, Wu Z, Ren J, Fan Y, Sun L, et al. Resurrection of endogenous retroviruses during aging reinforces senescence. Cell [Internet]. 2023;186:287–e30426. 10.1016/j.cell.2022.12.017. 36610399

[39] Halcrow PW, Quansah DNK, Kumar N, Steiner JP, Nath A, Geiger JD. HERV-K (HML-2) Envelope Protein Induces Mitochondrial Depolarization and Neurotoxicity via Endolysosome Iron Dyshomostasis. J Neurosci [Internet] Soc Neurosci. 2024;44:e0826232024. 10.1523/JNEUROSCI.0826-23.2024. [cited 2026 May 8];. PMC10993035 38383499

[40] Chabukswar S, Grandi N, Tramontano E. Prolonged activity of HERV-K(HML2) in Old World Monkeys accounts for recent integrations and novel recombinant variants. Front Microbiol [Internet]. 2022;13:1040792. 10.3389/fmicb.2022.1040792. PMC9751479 36532485

[41] Marsh AK, Willer DO, Skokovets O, Iwajomo OH, Chan JK, MacDonald KS. Evaluation of cynomolgus macaque (Macaca fascicularis) endogenous retrovirus expression following simian immunodeficiency virus infection. Le Grand R, editor. PLoS One [Internet]. 2012;7:e40158. 10.1371/journal.pone.0040158. PMC3387136 22768246

[42] Kraus B, Fischer K, Büchner SM, Wels WS, Löwer R, Sliva K, et al. Vaccination directed against the human endogenous retrovirus-K envelope protein inhibits tumor growth in a murine model system. Kozak CA, editor. PLoS One [Internet]. 2013;8:e72756. 10.1371/journal.pone.0072756. PMC3758348 24023643

[43] Hohn O, Hanke K, Bannert N. HERV-K(HML-2), the Best Preserved Family of HERVs: Endogenization, Expression, and Implications in Health and Disease. Front Oncol [Internet]. 2013;3:246. 10.3389/FONC.2013.00246. [cited 2026 Jun 3];. PMC3778440 24066280

[44] Tao H, Hu Y. Correlation of HERVK with efficacy on anti-PD-1/PD-L1 therapy in a pilot metagenomics study on Chinese advanced lung cancer patients. J Clin Oncol [Internet] Am Soc Clin Oncol. 2019;37:e14046–14046. 10.1200/JCO.2019.37.15_SUPPL.E14046. [cited 2026 May 10];.

[45] Andersson A-MC, Schwerdtfeger M, Holst PJ. Virus-like-vaccines against HIV. Vaccines (Basel) [Internet]. 2018;6:10. 10.3390/vaccines6010010. PMC5874651 29439476

[46] Morozov VA, Morozov AV, Semaan M, Denner J. Single mutations in the transmembrane envelope protein abrogate the immunosuppressive property of HIV-1. Retrovirology [Internet]. Retrovirology; 2012 [cited 2026 May 10];9. 10.1186/1742-4690-9-67. PMC3464125 22889273

[47] Bonaventura P, Alcazer V, Mutez V, Tonon L, Martin J, Chuvin N, et al. Identification of shared tumor epitopes from endogenous retroviruses inducing high-avidity cytotoxic T cells for cancer immunotherapy. Sci Adv [Internet]. 2022;8:eabj3671. 10.1126/sciadv.abj3671. PMC8791462 35080970

[48] van de Vaccaro A, Ramachandran M, Essand M, Dimberg A. Of mice and lymphoid aggregates: modeling tertiary lymphoid structures in cancer. Front Immunol [Internet] Front Immunol. 2023. 10.3389/FIMMU.2023.1275378. [cited 2026 Jun 3];14. PMC10639130 37954592

[49] Alspach E, Lussier DM, Miceli AP, Kizhvatov I, DuPage M, Luoma AM, et al. MHC-II neoantigens shape tumour immunity and response to immunotherapy. Nat [Internet]. 2019;574:696–701. 10.1038/s41586-019-1671-8. PMC6858572 31645760

[50] Vanhersecke L, Brunet M, Guégan J-P, Rey C, Bougouin A, Cousin S, et al. Mature tertiary lymphoid structures predict immune checkpoint inhibitor efficacy in solid tumors independently of PD-L1 expression. Nat Cancer [Internet]. 2021;2:794–802. 10.1038/s43018-021-00232-6. PMC8809887 35118423

[51] Zander R, Schauder D, Xin G, Nguyen C, Wu X, Zajac A, et al. CD4 + T Cell Help Is Required for the Formation of a Cytolytic CD8 + T Cell Subset that Protects against Chronic Infection and Cancer. Immun [Internet]. 2019;51:1028–e10424. 10.1016/j.immuni.2019.10.009. PMC6929322 31810883

[52] Morozov VA, Morozov AV. A Comprehensive Analysis of Human Endogenous Retroviruses HERV-K (HML.2) from Teratocarcinoma Cell Lines and Detection of Viral Cargo in Microvesicles. Int J Mol Sci [Internet] Int J Mol Sci. 2021. 10.3390/IJMS222212398. [cited 2026 May 10];22. PMC8619701 34830279

[53] Sacha JB, Kim I-J, Chen L, Ullah JH, Goodwin DA, Simmons HA, et al. Vaccination with Cancer- and HIV Infection-associated Endogenous Retrotransposable Elements is Safe and Immunogenic. Volume 189. Oxford University Press (OUP); 2012;1467. [cited 2026 Aug 23];. 10.4049/JIMMUNOL.1200079. PMC3401319 22745376

[54] Eksmond U, Jenkins B, Merkenschlager J, Mothes W, Stoye JP, Kassiotis G. Mutation of the putative immunosuppressive domain of the retroviral envelope glycoprotein compromises infectivity. Sundquist WI, editor. J Virol [Internet]. 2017;91. 10.1128/JVI.01152-17. PMC5640850 28814524

