# Supplementary material for: Engineering human endogenous retrovirus-K for improving immunogenicity and broad cancer targeting
Source: J Transl Med. 2026 Sep 24;24:1337. doi: 10.1186/s12967-026-09012-1 (PMC13693031; doi:10.1186/s12967-026-09012-1)
Supplement: Supplementary file 1 [file 12967_2026_9012_MOESM1_ESM.docx]

**Supplementary Data**

|  | **Frequency** |
| --- | --- |
| **Variant/Total** | 91 |
| AML | 2 |
| Adrenal cancer | 15 |
| Anal cancer | 2 |
| Appendiceal cancer | 6 |
| Biliary tract cancer | 37 |
| Brain cancer | 6 |
| Breast cancer | 223 |
| CUP | 27 |
| Cervical cancer | 24 |
| Colorectal cancer | 307 |
| Endometrial cancer | 16 |
| Esophageal cancer | 32 |
| Fallopian tube cancer | 1 |
| Gastric_GEJ cancer | 48 |
| Germ cell tumor | 2 |
| Head and neck cancer | 26 |
| Liver cancer | 11 |
| Lymphoma non-Hodgkin | 4 |
| MANEC | 1 |
| Melanoma | 25 |
| Mesothelioma | 30 |
| MiNEN | 7 |
| NEC | 20 |
| NET | 3 |
| NSCLC | 138 |
| NUT carcinoma | 2 |
| Other | 11 |
| Ovarian cancer | 51 |
| Pancreatic cancer | 44 |
| Penile cancer | 4 |
| Peritoneal cancer | 1 |
| Prostate cancer | 66 |
| Pseudomyxoma peritonei | 1 |
| Renal cell carcinoma | 5 |
| SCLC | 30 |
| Salivary gland cancer | 8 |
| Sarcoma | 18 |
| Skin cancer - non-melanoma | 2 |
| Small intestine cancer | 3 |
| Testicular cancer | 6 |
| Thymic carcinoma | 13 |
| Thymoma | 6 |
| Thyroid cancer | 4 |
| Urothelial cancer | 24 |
| Vaginal cancer | 1 |

**Supplementary Figure S1.**

CoPPO cohort composition. Number of patients represented in each of the cancer types, cancers of unknown primary (CUP), and unclassified cancers (“Other” and “Unspecified”).

**Supplementary Figure S2.**

Predicted HLA class I strong binder load of HERV-K (HML-2) GAG and ENV proteins relative to HERV-K-ISDmut immunogen. Each dot represents a genomic HERV-K provirus (RepeatMasker HERVK-int) grouped on the X axis by coding class assigned from conserved retroviral protein domains (HMM/GyDB annotation): loci with intact GAG domain only (n=20), ENV domain only (n=33) or both (n=41). All amino acid sequence domains were screened with NetMHCpan-4.1 for binding to 20 HLA class I alleles using 9-mer peptides. The Y axis shows the number of predicted strong binders (SB; %Rank_EL < 0.5) summed across all gag and env ORFs at the locus and across all 20 alleles. Black marks are the class median. Dots are coloured by their identity to HERV-K-ISDmut computed as the length-weighted mean BLASTp percent identity. Loci with >90% identity to HERV-K-ISDmut are outlined in red.

**Supplementary Figure S3.**

Fusion activity of HERV-K ENV when co-expressed with HERV-K GAG or expressed alone. Error bars represent SEM. The experiment was performed 3 times with 5-6 biological replicates.

**Supplementary Figure S4.**

Using Ad19a-HERV-K-ISDmut in matured human moDCs gives the highest HERV-K transgene expression. Healthy human donor monocytes isolated from PBMCs were differentiated to moDCs using a commercial differentiation supplement and differentiation was verified by CD11a staining. Then the cells were either matured using a commercial maturation supplement or left immature. Maturation was verified using CD80, CD83 and CD86 staining. Cells were transduced with an Ad19a vector containing either the HERV-K-WT or the HERV-K-ISDmut and transduction efficacy was tested using an Ad19a vector containing GFP. Top: shows the transgene HERV-K expression as fraction of total live cell counts at 28, 50 and 70 hours. Consistently throughout optimization, the biggest difference in transgene expression between HERV-K-WT and HERV-K-ISDmut was seen at roughly 48 hours in immature DCs. The highest expression levels were observed at roughly 24 and 48 hours in matured moDCs. Bottom: shows moDC viability over time. The HERV-K insert had a higher impact on viability loss than did the adenoviral vector itself. Maturation also decreases viability.

**Supplementary Figure S5.**

Higher transgene (consensus) HERV‑K expression in paired samples was observed in WT transduced human moDCs. Boxplots show normalized HERV‑K mapped read counts (CPM) for the two vector conditions (WT and ISD) measured in the same donors (paired design). Individual donor measurements are overlaid as points and labeled (Donor 2, 4, 5, 6, 7, 8). Statistical significance was assessed using a paired t‑test on log2(CPM+1) and a paired sign‑flip permutation test on the mean log2-difference; reported p-values are *P* = 0.0013 (paired t‑test) and *P* = 0.0615 (permutation).

**C**

| **panel** | **PC** | **var_explained_%** | **donor_ANOVA_P** | **vector_paired_t_P** | **vector_Wilcoxon_P** | **lib_size_rho** | **lib_size_P** | **RIN_rho** | **RIN_P** | **conc_ng_ul_rho** | **conc_ng_ul_P** |
| --- | --- | --- | --- | --- | --- | --- | --- | --- | --- | --- | --- |
| **A (unadjusted, n=11)** | PC1 | 58.9 | 0.00011 | 0.01370 | 0.0625 | -209 | 0.53722 | 709 | 0.01455 | -991 | 3.76257e-09 |
| **A (unadjusted, n=11)** | PC2 | 16.1 | 3.11235e-08 | 0.20344 | 0.4375 | 0.3 | 0.37008 | 0.5 | 0.11730 | -145 | 0.66957 |
| **A (unadjusted, n=11)** | PC3 | 9.0 | 4.99605e-05 | 0.00586 | 0.0625 | -309 | 0.35502 | -164 | 0.63068 | 309 | 0.35502 |
| **B (donor-adjusted, 5 pairs)** | PC1 | 63.6 | 1.0 | 4.41091e-05 | 0.0625 | -55 | 0.88103 | 333 | 0.34659 | 115 | 0.75141 |
| **B (donor-adjusted, 5 pairs)** | PC2 | 12.9 | 1.0 | 0.88379 | 1.0 | 164 | 0.65147 | 42 | 0.90736 | 42 | 0.90736 |
| **B (donor-adjusted, 5 pairs)** | PC3 | 8.9 | 1.0 | 0.96736 | 1.0 | -67 | 0.85481 | -0.03 | 0.93377 | 42 | 0.90736 |

**Supplementary Figure S6.**

Bulk RNA sequencing of human monocyte-derived dendritic cells from six healthy donors (libraries analyzed in Fig 3D and 3E). **A,** All 11 sequenced libraries. Color denotes vector, symbol denotes donor. Samples cluster by donor rather than vector. PC1 (58.9% of total variance) and PC2 (16.1%) are both associated with donor (one-way ANOVA P = 1.1 × 10−4 and P = 3.1 × 10−8), which accounts for 98.5% and 99.9% of their respective sample-level variance against 3.3% and 0.1% for vector. **B**, The five donors with a complete WT/ISDmut pair, after removal of the donor term (limma::removeBatchEffect). The two vectors separate along PC1, which carries 63.6% of the residual variance and is 98.9% explained by vector (paired t-test on PC1 scores, P = 4.4 × 10−5; Wilcoxon signed-rank P = 0.063, the minimum attainable with five pairs). **C,** Association of principal components with biological and technical covariates. For the unadjusted principal component analysis (11 libraries) and the donor-adjusted analysis (five complete donor pairs) shown in **A** and **B**, PC1–PC3 were tested against donor (one-way ANOVA), vector (paired t-test and Wilcoxon signed-rank test across donor pairs) and three technical covariates (total mapped library size, RNA integrity number, and RNA input concentration measured by Nanodrop; Spearman correlation). In the unadjusted analysis, PC1 is strongly associated with RNA input concentration (ρ = −0.99, P = 3.8 × 10−9) and with RNA integrity number (ρ = 0.71, P = 0.015). Both are donor-level properties in this experiment (concentrations cluster tightly within donor and span an approximately eight-fold range between donors) and are therefore collinear with donor. After removal of the donor term, PC1 shows no association with library size (ρ = −0.06, P = 0.88), RNA integrity number (ρ = 0.33, P = 0.35) or RNA input concentration (ρ = 0.12, P = 0.75), while the association with vector becomes highly significant (P = 4.4 × 10−5). PC2 and PC3 of the donor-adjusted analysis show no association with any variable tested (all P > 0.65).

 **Supplementary Figure S7.**

Gating strategy used for analysis of viability and transgene expression in immature DCs.

**Supplementary Figure S8.**

Graphic showing the principle used in T cell expansion experiments. Anonymized buffy coats were obtained from Rigshospitalet, Denmark and subsequently isolated and frozen. Aliquots were thawed and processed at the above outlined timepoints to achieve co-cultures of donor PBMCs with pre-differentiated, matured and transduced autologous moDCs. The initial 2 co-cultures were with Ad19a transduced moDCs while the last 2 co-cultures were with Ad5F35 transduced moDCs to decrease the stimulation toward the same adenoviral vector. IL-2 was added to the cultures at only 48h prior or post co-culture. After 14 days of culture PBMCs were stimulated with HERV-K WT consensus sequence peptide pools 1-4 as described in materials and methods. After incubation, cells were labelled with anti-IFN-γ antibodies and sorted with magnetic beads. The sorted cells were expanded using IL-2, anti-CD3, and irradiated ”feeder cells” (a mix of 3 other donors unsorted PBMCs). Analysis of responses were conducted using individual peptide pool 1-4 stimulation, intracellular staining, and measuring by flow cytometry. Created with BioRender.com.

 **Supplementary Figure S9.**

Stimulation with mature human moDCs can expand human T cells e*x vivo* toward full length HERV-K GAG and ENV proteins. Showing both measured timepoints otherwise seen in Fig. 4, A and B. Human donor PBMCs stimulated *ex vivo* with either HERV-K-WT or HERV-K-ISDmut using transduced autologous moDCs. HERV-K-ISDmut stimulated cells consistently tended to show HERV-K recognition at higher frequencies and across more targets, especially for Gag pools. As observed for the measurements on day 29, ISDmut stimulated cells tend to exhaust less.

 **Supplementary Figure S10.**

Gating strategy used for analysis of human T cell responses upon restimulation with HERV-K WT consensus sequence peptide pools 1-4.

**Supplementary Figure S11.**

Polyclonal anti-HERV-K serum was generated in rats. Outbred rats were primed at day 0 with 2 × 10^8^ IFU Ad19a-HERV-K ISDmut s.c. and boosted on day 28 with 2 × 10^8^ Ad5F35-HERV-K ISDmut IFU s.c. The control group was immunized with 2 × 10^8^ IFU Ad19a-CTRL (empty vector) s.c. on day 0 and 2 × 10^8^ IFU Ad5F35-CTRL (empty vector) s.c. on day 28. Serum was harvested on day 56 for analysis of responses against the HERV-K ENV surface unit (SU) protein by ELISA. Serum harvested on day 56 was used for flow cytometry in Figure 5, G and H and Supplementary FigureS12.

**Supplementary Figure S12.**

Polyclonal rat anti-HERV-K antibodies stain human cancer cell lines. Protein was isolated from the cell pellets of cancer cell lines and analyzed by western blot using HERV-K rat serum or control serum generated as shown in Supplementary Figure S11.

**Supplementary Figure S13.**

HERV-K transgenic mice were generated and selected. **A,** Schema illustrating the generation of HERV-K C57BL/6 transgenic mice (created with BioRender.com). Pronuclear injection of the DNA transgene was performed into fertilized oocyte. The transgenic embryo was then transferred into a pseudo pregnant C57BL/6 mouse. Transgenic offspring were crossed with wild type C57BL/6 mice to generate both transgenic and wild type mice. Based on the magnitude of the HERV-K specific T cell responses, the transgenic mouse founder was selected to be bred with Balb/C mice and generate CB6F1 transgenic and wild type mice. The transgene encoded for the MMTV LTR promoter, HERV-K Gag and Env and the Tet-on system. SU, surface unit; TM, transmembrane unit; CT, cytoplasmic tail. **B,** pUC57 DNA plasmid map including the HERV-K transgene that was inserted into the fertilized oocyte. **C,** The schema on top illustrates the immunization schedule of the C57BL/6 transgenic mice and their wild type littermates generated from three different transgenic founder offspring (#A, #B and #C, created with BioRender.com). Mice (n = 2-4 per group) were immunized with 2 × 10^8^ IFU Ad19a-HERV-K-ISDmut. The frequency of IFN-γ producing CD44^hi^ CD8^+^ T cells responding to the HERV-K pool 2 Gag (peptides 63-133) was evaluated by ICS from the splenocytes of mice 21 days after the immunization (bottom graph).

**Supplementary Figure S14.**

No significant differences observed in anti HERV-K immunity *in vivo* between Ad5F35 and Ad5 vectors. **A,** CB6F1 WT (WT) littermates and CB6F1 HERV-K Transgenic (Tg) mice (generated as in fig. S12) were primed on day 0 with 2 × 10^8^ IFU Ad19a-HERV-K-ISDmut s.c. (hock) and boosted on day 28 with 2 × 10^8^ IFU Ad5-HERV-K-ISDmut s.c. (hock). Anti-PD-1 or isotype control (Iso-CTRL), 200 µg, was administered i.p. on day 0, 7, 14, and day 28 or day 3, 7, 14, and day 28. Spleens were harvested on day 38 and splenocytes were stimulated with the envelope specific peptide 18 and stained intracellularly. Analysis was done by flow cytometry. Blood was harvested on day 14, 28 and 38, and T cells were stained by tetramer staining with envelope specific peptide 18. Blood was harvested on day 0 and day 38, serum isolated and analyzed for responses against HERV-K envelope surface unit (SU) by ELISA. **B,** CB6F1 WT (WT) littermates and CB6F1 HERV-K Transgenic (Tg) mice (generated as in supplementary figure 9) were primed on day 0 with 2 × 10^8^ IFU Ad19a-HERV-K-ISDmut s.c. (hock) and boosted on day 28 with either 2 × 10^7^ IFU Ad5-HERV-K-ISDmut s.c. (hock) or 2 × 10^7^ IFU Ad5F35-HERV-K-ISDmut s.c. (hock). Anti-PD-1, 200 µg, was administered i.p. on day 0, 8, 15, and day 28. Spleens were harvested on day 69 and splenocytes were either stimulated with envelope specific peptide 18 and stained intracellularly, or tetramer staining was performed with a tetramer bound by the same peptide 18. Analysis was done by flow cytometry. Blood was harvested on day 0 and day 69, serum isolated and analyzed for responses against HERV-K envelope surface unit (SU) by ELISA. **P* < 0.05, ***P* < 0.01, ****P* < 0.001, and *****P* < 0.0001 by two-way ANOVA with Tukey's multiple-comparison test.
